# A multiplatform approach identifies miR-152-3p as a common epigenetically regulated onco-suppressor in prostate cancer targeting *TMEM97*

**DOI:** 10.1186/s13148-018-0475-2

**Published:** 2018-03-27

**Authors:** João Ramalho-Carvalho, Céline S. Gonçalves, Inês Graça, David Bidarra, Eva Pereira-Silva, Sofia Salta, Maria Inês Godinho, Antonio Gomez, Manel Esteller, Bruno M. Costa, Rui Henrique, Carmen Jerónimo

**Affiliations:** 1 Cancer Biology & Epigenetics Group – Research Center (CI-IPOP), Portuguese Oncology Institute of Porto (IPO Porto), F Bdg, 1st floor, Rua Dr António Bernardino de Almeida, 4200-072 Porto, Portugal; 2 Cancer Epigenetics and Biology Program, Bellvitge Biomedical Research Institute, Barcelona, Catalonia Spain; 3 Biomedical Sciences Graduate Program, Institute of Biomedical Sciences Abel Salazar– University of Porto (ICBAS-UP), Porto, Portugal; 4 Life and Health Sciences Research Institute (ICVS), School of Medicine, University of Minho, Campus de Gualtar, Braga, Portugal; 5 ICVS/3B’s - PT Government Associate Laboratory, Braga/Guimarães, University of Minho, Campus de Gualtar, Braga, Portugal; 6 Department of Immunology, Portuguese Oncology Institute of Porto, Porto, Portugal; 7 Institucio Catalana de Recerca i Estudis Avançats (ICREA), Barcelona, Catalonia Spain; 8 Physiological Sciences Department, School of Medicine and Health Sciences, University of Barcelona (UB), Barcelona, Catalonia Spain; 9 Department of Pathology, Portuguese Oncology Institute of Porto, Porto, Portugal; 10 Department of Pathology and Molecular Immunology, Institute of Biomedical Sciences Abel Salazar– University of Porto (ICBAS-UP), Porto, Portugal; 11 Present Address: Division of Oncogenomics, Netherlands Cancer Institute, Plesmanlaan 121, 1066 CX Amsterdam, the Netherlands; 12 Present Address: Gene Regulation, Stem Cells and Cancer Programme, Centre for Genomic Regulation (CRG), The Barcelona Institute of Science and Technology, Barcelona, Spain

**Keywords:** miR-152-3p, Prostate cancer, Cell cycle, DNA methylation, CRISPR, *TMEM97*

## Abstract

**Background:**

Prostate cancer (PCa) is a major cause of morbidity and mortality in men worldwide. MicroRNAs are globally downregulated in PCa, especially in poorly differentiated tumors. Nonetheless, the underlying mechanisms are still elusive. Herein, using combined analysis of microRNAs expression and genomewide DNA methylation, we aimed to identify epigenetically downregulated microRNAs in PCa.

**Results:**

We found that miR-152-3p was underexpressed in PCa and that lower expression levels were associated with promoter hypermethylation in accordance with TCGA dataset analysis. Functional in vitro assays suggest that miR-152-3p suppresses cell viability and invasion potential, whereas it promotes cell cycle arrest at S and G2/M phases. Additionally, miR-152-3p expression was associated with longer disease-free survival in PCa patients from TCGA. Finally, *TMEM97*, which is overexpressed in PCa, was identified as a novel miR-152-3p target gene.

**Conclusions:**

Our findings demonstrate the advantages of using a combinatory approach to identify microRNAs downregulated due to aberrant promoter methylation. MiR-152-3p downregulation and promoter methylation was found to be prevalent in primary PCa, which impairs its role in control of cell viability, cell cycle regulation and invasion.

**Electronic supplementary material:**

The online version of this article (10.1186/s13148-018-0475-2) contains supplementary material, which is available to authorized users.

## Background

MicroRNAs (miRNAs) are a class of small (~ 22-nucleotide) RNAs that mediate post-transcriptional gene silencing by guiding Argonaute (AGO) proteins to target mRNAs [1, 2], either by repressing translation or by promoting destabilization [3]. Target guidance and specificity is mainly determined by nucleotides at positions 2–7 of the miRNA (the seed-sequence) [4]. Such mechanisms are critical for homeostasis maintenance, both under physiological conditions and in cell’s response to environment alterations, including stress signals [5]. Thus, a vast number of biological processes are subject to miRNA-dependent regulation, encompassing cell proliferation, signaling, differentiation, stress response, DNA repair, cell adhesion and motility, inflammation, cell survival, senescence, and apoptosis [1]. Interestingly, miRNA’s expression, processing, and functional output are also stringently controlled [6]. Indeed, miRNAs’ expression and activity are tightly spatially and temporally regulated, and its disruption has been extensively linked to human disease, including the development of cancer and metastasis formation [1, 7]. Globally, miRNAs are mostly downregulated in cancer, including that of the prostate [1]. Multiple mechanism are known to induce miRNA deregulation, including epigenetic alterations, aberrant transcription factors binding (e.g., p53, MYC), miRNA biogenesis machinery disruption, RNA editing, post-transcriptional RNA modifications, Argonaute loading, and RNA decay [7, 8].

Prostate cancer (PCa) is the most common non-cutaneous cancer worldwide in men, and a leading cause of cancer-related death in developed countries [9]. Multiple factors, comprising age, family history, genetic susceptibility and ethnicity, contribute to the high incidence of PCa [10]. Owing to its complexity and heterogeneity, and despite extensive studies, the molecular mechanisms that drive prostatic carcinogenesis are still far from complete understanding. Because miRNAs regulate a wide range of signaling pathways that are frequently deregulated in PCa, this class of noncoding RNAs might be of critical relevance for tumor development and progression. Thus, its study may provide novel insights into PCa biology and afford innovative tools for patient management, aiding in diagnosis and prognosis assessment, as well as the identification of new therapeutic targets [11].

## Methods

### Aim

Here, we attempted to discover new epigenetically regulated miRNA loci in PCa using a combinatory approach that compared miRNAs expression profiling with DNA methylation patterns. The candidate microRNAs were subsequently validated in two large patient cohorts, which included ours and that of TCGA; in vitro assays were performed to characterize their role in cancer cell biology, and in silico analysis, followed by in vitro validation, allowed for the identification of relevant target mRNAs. Overall, our data extends current knowledge about epigenetic deregulation and biological significance of miRNAs in prostate carcinogenesis. A flow chart depicting the different steps followed in this study is provided in Additional file 1: Figure S1. All methods were performed in accordance with the relevant guidelines and regulations both for tissue samples and in vitro assays.

### Patient and samples

PCa tissue samples (*n* = 100) from patients diagnosed and primarily treated with radical prostatectomy at Portuguese Oncology Institute of Porto, Portugal, were prospectively collected. Fourteen normal prostate tissue (MNPT) samples, of peripheral zone of prostates without PCa, from patients submitted to radical cystoprostatectomy due to bladder cancer, served as controls. All specimens, promptly frozen at − 80 °C, were cut for nucleic acid extraction. For routine histopathological examination, formalin-fixed and paraffin-embedded (FFPE) fragments were also collected. Relevant clinical data was retrieved from clinical charts. This study was approved by the institutional ethics committee [Comissão de Ética para a Saúde- Instituto Português de Oncologia do Porto Francisco Gentil, EPE (CES-IPOPFG-EPE 215/013)]. Moreover, in compliance with the Helsinki Declaration and after CES approval, informed consent was obtained for all patients previously to surgery. Additionally, a Cohort of patients available at TCGA were included for validation. The clinical and pathological data of both cohorts of patients (IPO Porto’s cohort and TCGA’s cohort) included in this study is reported in Table 1.

**Table 1:** Clinical and pathological data of the patients included in this study

(A) IPO Porto’s cohort		
Clinicopathological features	MNPT	PCa
Patients, *n*	14	100
Median age, years (range)	65 (49–80)	65 (49–75)
PSA (ng/mL), median (range)	n.a.	8.45 (3.5–23)
pT2	n.a.	60 (60%)
pT3	n.a.	40 (40%)
< 7	n.a*.*	36 (36%)
= 7	n.a.	58 (58%)
> 7	n.a.	6 (6%)
(B) TCGA’s cohort		
Clinicopathological Features	NAT	PCa
Patients, n	52	497
Median age, years (range)	61 (43–72)	61 (41–78)
pT2	29 (56%)	189 (38%)
pT3	21 (40%)	292 (59%)
pT4	2 (4%)	10 (2%)
< 7	5 (10%)	45 (9%)
= 7	40 (77%)	248 (50%)
> 7	7 (13%)	204 (41%)

*MNPT* morphologically normal prostate tissue, *PCa* prostate cancer, *NAT* normal adjacent tissue, *n.a.* not applicable

(A) IPO Porto’s cohort (B) TCGA’s cohort

### PCa cell lines and demethylation treatment

Prostate cell lines, LNCaP, 22RV1, DU145, PC-3 (malignant), and RWPE (benign) were used for in vitro studies. LNCaP and 22Rv1 cells were grown in RPMI 1640, whereas DU145 and PC-3 cells were maintained in MEM and 50% RPMI-50% F-12 medium, while RWPE was cultured in Keratinocyte-SFM, containing human recombinant Epidermal Growth Factor 1-53 and Bovine Pituitary Extract (GIBCO, Invitrogen, Carlsbad, CA, USA), respectively. HEK293Ta were maintained in DMEM. All basal culture media were supplemented with 10% fetal bovine serum and 1% penicillin/streptomycin (GIBCO, Invitrogen, Carlsbad, CA, USA). Cells were maintained in an incubator at 37 °C with 5% CO_2_. All cell lines were G-banding karyotyped (for validation) and routinely tested for *Mycoplasma spp*. contamination (PCR Mycoplasma Detection Set, Clontech Laboratories).

One micromolar of the DNA methyltransferases inhibitor 5-aza-2-deoxycytidine (5-Aza-CdR; Sigma-Aldrich, Schnelldorf, Germany) was used for DNA demethylation. Cells were harvested and RNA extracted after 72-h exposure to the demethylating agent.

### Nuclei acid extraction and bisulfite conversion

DNA was extracted from fresh frozen tissue samples and cell lines using phenol: chloroform (Sigma). RNA was isolated using TRIzol (Invitrogen, Carlsbad, CA, USA) according to the manufacturer’s instructions.

Bisulfite conversion of genomic DNA (1000 ng) was accomplished using EZ DNA Methylation Kit (Zymo Research), following the manufacturer’s instructions.

### sgRNA cloning

Complementary single-stranded oligos (Additional file 1: Table S1) were phosphorylated and annealed by combining 100 μM oligos, 1× T4 PNK Buffer, 1 mM ATP, 5 U T4 PNK and incubating the reaction at 37 °C/30 min, 95 °C/5 min followed by a ramp down to 25 °C at 5 °C/min. Annealed oligos were diluted at 1:100 in sterile water and ligated to plasmid vector lentiCRISPRv2 (gift from Feng Zhang (Addgene plasmid #52961)) using the following parameters: 50 ng BsmBI (Fermentas) digested plasmid, 1 μl diluted oligo duplex, 1× Ligation Buffer (Roche), and 5 U T4 DNA Ligase (Roche) incubated at RT/30 min. The ligation reactions were used to transform highly competent *Escherichia coli* cells according to the manufacturer’s protocol [12]. Transformation mixtures were plated in LB-agar plates. After colony selection, they grew in liquid LB and plasmid DNA was harvested using PureLink HiPure Plasmid Maxiprep Kit (Invitrogen, Carlsbad, CA, USA). The resulting DNA was then subjected to Sanger sequencing to confirm the correct either the orientation and sequence of each sgRNA.

### Lentivirus production, purification, and transduction

To produce lentivirus, 4 × 10^6^ HEK293T cells per sgRNA were seeded in ten 100-mm dishes 1 day before transfection. For each dish, we diluted 10 μg of plasmid DNA (corresponding to individual sgRNA), 3.5 μg of pVSV-G, 5 μg of pMDL RRE, and 2.5 μg of pRSV-REV in 450 μl of 0.1× TE/H2O, added 50 μl of CaCl2 and incubated 5 min at RT. Plasmid DNA was precipitated by adding 500 μl 2× HBS to the solution while vortexing at full speed. The precipitate was added immediately to the plate and the cells were incubated for 14 h at 37 °C, after which the medium was refreshed. Lentivirus-containing supernatants were collected 60 h post-transfection, filtered through a 0.45-μm membrane (Milipore Steriflip HV/PVDF) and stored at − 80 °C. Cell lines were infected with lentivirus supernatants supplemented with 8 μg/ml polybrene (Sigma). At 24 h post-infection, medium was replaced and cells were selected with 2 μg/ml puromycin (Gibco). Antibiotic selection was stopped as soon as no surviving cells remained in the no-transduction control plate.

### PCR and sanger sequencing

Genomic DNA (∼ 1 × 10^5^ cells) from cloned cells was isolated with DNeasy Blood and Tissue kit (Qiagen). PCR reactions were carried out with 500 ng of genomic DNA using Phusion DNA polymerase (Thermo Scientific) according to the manufacturer’s instructions. The PCR products were run in a gel and purified using the Agarose Gel DNA Extraction Kit (Roche). The primer pairs spanning the target site (covering around 500 bp for each cutting site) are listed in the Additional file 1: Table S1. Purified PCR samples (50 ng) were prepared for sequencing using 4 μl of BigDye terminator v3.1 (Applied Biosystems) and 5 pM primer in final volume of 20 μl. PCR program: 1 min at 96 °C (1×), followed by 30 s at 96 °C, 15 s at 50 °C, and 4 min at 60 °C (30×), and finishing with 1 min incubation at 4 °C (1×). Samples were analyzed in an Applied Biosystems 3730xl DNA Analyser. The quantitative assessment of CRISPR-Cas9 genome editing was done using a freely available online software—TIDE [13]. Specifically, using Sanger sequencing reactions (sgRNA NT, sgTMEM97#1.1, sgTMEM97#1.2, sgTMEM97#2.1 and sgTMEM97#2.2), insertions/deletions (indels) and editing efficacy was assessed by TIDE software [13]. For that, the chromatogram sequence files of respectively the control sample (i.e., transduced with the sgRNA NT) and the test sample (i.e., transduced with the target sgRNA) were analyzed. As output, the quantitative spectrum of indels around the cut site was obtained [13].

### MicroRNA expression profiling

MiRNAs expression was assessed in ten PCa and four MNPT using microRNA Ready-to-Use PCR Human Panel (I + II) v2.R (Exiqon, Vedbaek, Denmark), comprising 752 miRNAs as previously described [14, 15]. Extracted RNAs were submitted to cDNA synthesis using miRCURY LNA Universal RT microRNA PCR (Exiqon, Vedbaek, Denmark) following the manufacturer’s instructions. Data were analyzed using the comparative Ct method, and the median value was calculated for reference genes’ expression normalization. MiRNAs with fold change of − 1.5 in PCa compared with MNPT were considered downregulated.

### MicroRNA’s promoter methylation analysis in prostate tissues

All DNA samples were assessed for integrity, quantity, and purity by electrophoresis in a 1.3% agarose gel, picogreen quantification, and nanodrop measurements. All samples were randomly distributed into 96-well plates. Bisulfite conversion of 500 ng of genomic DNA was performed using the EZ DNA Methylation Kit (Zymo Research) following the manufacturer’s instructions. Two hundred nanograms of bisulfite-converted DNA was used for hybridization on the HumanMethylation450 BeadChip (Illumina). Briefly, samples were whole-genome amplified followed by enzymatic end-point fragmentation, precipitation, and resuspension. The resuspended samples were hybridized onto the BeadChip for 16 h at 48 °C, then washed. A single nucleotide extension with labeled dideoxynucleotides was performed, and repeated rounds of staining were applied with a combination of labeled antibodies differentiating between biotin and DNP.

HumanMethylation450 BeadChip data were processed using Bioconductor minfi package [16]. The “lllumina” procedure that mimics the method of GenomeStudio (Illumina) was performed, including background correction and normalization considering the first array of the plate as reference. Probes with one or more single nucleotide polymorphisms (SNPs) with a minor allele frequency (MAF) > 1% (1000 Genomes) in the first 10 bp of the interrogated CpG were removed. The methylation level (β value) for each of the 485,577 CpG sites was calculated as the ratio between the methylated probe intensity and the overall intensity (sum of methylated and unmethylated probe intensities) multiplied by 100. After normalization step, probes mapped within X and Y chromosomes were removed. All analyses were performed in human genome version 19 (hg19), and data was deposited in GEO repository under accession number GSE52955.

### TCGA dataset analysis

Data on miRNA expression and clinical information (when available) from PCa and matched normal tissue samples was retrieved from The Cancer Genome Atlas (TCGA) database. The mRNA expression data from samples hybridized at University of North Carolina, Lineberger Comprehensive Cancer Center, using Illumina HiSeq 2000 mRNA Sequencing version 2, were downloaded from data matrix including 494 miRNA-Seq, 496 RNA-Seq, and 498 Methylation Array for PCa samples and 52 matched normal adjacent tissue samples (NAT). To prevent duplicates, when there was more than one portion per patient, median values were used. The provided value was pre-processed and normalized according to “level 3” specifications of TCGA. Clinical data of each patient was provided by Biospecimen Core Resources (BCRs). Data is available for download through https://gdc-portal.nci.nih.gov/projects/TCGA-PRAD.

### Real-time quantitative PCR (RT-qPCR)

MiR-152-3p transcript levels were assessed using TaqMan MicroRNA Assay (assay ID: 000475; Applied Biosystems) and normalized with RNU48 (assay ID: 001006; Applied Biosystems).

Real-time quantitative PCR analysis was performed using gene-specific primers and normalized using *GUSB* housekeeping gene (Additional file 1: Table S1). Specific-miRNA cDNA was obtained using TaqMan MicroRNA Reverse Transcription Kit from Applied Biosystems (Foster City, CA, USA). Total cDNA synthesis was performed using high-capacity cDNA Reverse Transcription Kit (Applied Biosystems, Foster City, CA, USA).

*NOL4* and *TMEM97* mRNA levels were confirmed in the same group of tissue samples previously indicated. A total of 300 ng was reverse transcribed and amplified using TransPlex® Whole Transcriptome Amplification Kit (Sigma-Aldrich®, Schnelldorf, Germany) with subsequent purification using QIAquick® PCR Purification Kit (QIAGEN, Hilden, Germany), according to the manufacturer’s instructions. Expression levels were evaluated using TaqMan® Gene Expression Assays (Applied Biosystems, Foster City, CA, USA), and GUSB was used as a reference gene for normalization.

The expression of each gene or small RNA was obtained using the formula: Relative expression = (Target gene mean quantity/Reference gene mean quantity). Ratios were then multiplied by 1000 for easier tabulation. Each plate included multiple non-template controls, and serial dilutions (× 10) of a cDNA obtained from human prostate RNA (Carlsbad, CA, USA) were used to construct a standard curve for each plate. All experiments were run in triplicates (Additional file 1: Table S1).

### DNA methylation analysis

DNA methylation analysis was performed by quantitative methylation PCR (qMSP) using KAPA SYBR FAST qPCR Kit (Kapa Biosystems, MA, USA) and pyrosequencing. All reactions were run in triplicates in 384-well plates using Roche LightCycler 480 II, with *β-actin* (*ACTB*) as internal reference gene for normalization. Primer sequences (Additional file 1: Table S1) were designed using Methyl Primer Express 1.0 and purchased from Sigma-Aldrich (St. louis, MO, USA).

For pyrosequencing, specific sets of primers for PCR amplification and sequencing were designed using a specific software pack (PyroMark assay design version 2.0.01.15). Primer sequences were designed, when possible, to hybridize with CpG-free sites to ensure methylation-independent amplification. PCR was performed under standard conditions with biotinylated primers, and the PyroMark Vacuum Prep Tool (Biotage, Uppsala, Sweden) was used to prepare single-stranded PCR products, according to the manufacturer’s instructions. Pyrosequencing reactions and methylation quantification were performed in a PyroMark Q96 System version 2.0.6 (Qiagen, Hilden, Germany) using appropriate reagents and recommended protocols (Additional file 1: Table S1).

### Pre-miR transfections

To overexpress miR-152-3p, synthetic, commercially available, miRNAs’ precursors (pre-miR-152-3p, ID: PM12269; pre-miR-NC, ID: AM17110; Ambion, Carlsbad, CA, USA) were transfected at 30 nM. Transfections were performed using Oligofectamine (Invitrogen, Carlsbad, CA, USA), per manufacturer’s instructions.

### Viability assay

Cell viability was evaluated by MTT assay. Briefly, PCa cells were seeded onto 96-well flat-bottomed culture plates, allowed to adhere overnight later (number of cells plated before transfection: LNCaP: 10000 cells/well; PC3: 3000 cells/well), and transfected 24 h later. At each time point, 0.5 mg/ml of MTT reagent [3-(4, 5dimethylthiazol-2-yl)-2, 5-diphenyl-tetrazolium bromide] was added to each well, and the plates were incubated in the dark for 1 h at 37 °C. Formazan crystals were then dissolved in DMSO and absorbance was read at 540 nm in a microplate reader (FLUOstar Omega, BMG Labtech, Offenburg, Germany), subtracting the background, at 630 nm. The number of cells was calculated using the formula: [(OD experiment x Number of cells at day 0)/Mean OD at day 0]. Three replicates were performed for each condition and at least three independent experiments were carried out.

### GFP-competitive proliferation assay

LNCaP cells were infected with sgRNAs targeting the exon 1 or exon 2 of *TMEM97*. Separately, we generated polyclonal LNCaP cells stably expressing GFP using pLX304-GFP30 (gift from David Root; Addgene plasmid # 25890). GFP expressing cells were mixed in a 1:3 ratio with cells containing individual sgRNAs. The percentage of GFP-expressing cells was assessed by flow cytometry at the beginning of the experiment (*T* = 0) and every 72 h onwards (*T* = 3d; *T* = 6d, and *T* = 9d). For each condition, 10,000 events were recorded. The cells were measured on a BD FACSCalibur cytometer (BD Biosciences, San Jose, CA, USA) and analyzed using FlowJo software.

### Apoptosis evaluation

Evaluation of apoptosis was performed using APOPercentage apoptosis assay kit (Biocolor Ltd., Belfast, Northern Ireland) according to the manufacturer’s instructions. PCa cells were seeded onto 24-well plates (LNCaP: 50000 cells/well, and PC3: 30000 cells/well) and 24 h later were transfected. Apoptotic cells were assessed at the end of the day 3, in a FLUOstar Omega microplate reader at 550 nm and the background subtracted at 620 nm. The results were normalized to number of viable cell obtained in the MTT assay according to the following formula (OD of apoptosis assay at 72 h/ OD of MTT at 72 h).

### Cell cycle analysis

Cell cycle distribution of LNCaP and PC3 cells was determined by flow cytometry. Briefly, 72 h after transfections, 5 × 10^5^ harvested cells were fixed overnight at 4 °C with 70% cold ethanol. After cold PBS washing, cells were re-suspended in staining Propidium Iodide Solution (Cytognos S.L, Salamanca, Spain) and incubated for 30 min at room temperature. All cells were then measured on a Cytomics FC500 flow cytometer (Beckman Coulter, Fullerton, CA, USA) and analyzed using Modfit LT (Verity Software House, Inc., Topshan, Maine, USA).

### Cell invasion assay

Cell invasion was determined using BD BioCoat Matrigel Invasion Chamber (BD Biosciences, Franklin Lakes, NJ, USA). Briefly, 5 × 10^4^ cells/mL of LNCaP or PC3 cells were added to the upper chamber. Both cell lines were transfected for 72 h with miRNA molecules, after which, the non-invading cells were removed with cotton swabs from the upper side of the membrane. The membrane bottom containing invading cells was fixed in methanol, washed in PBS, and stained with DAPI (Vector Laboratories, Burlingame, CA). All the invading cells were counted under a fluorescent microscope. PC3 cell line invasion capability was quantified upon trans-well matrigel invasion assays (*n* = 3 for each sample) comparing pre-miR-152-3p to pre-miR-NC cells. Error bars in all panels indicate standard deviation, unless otherwise specified.

### Transcriptomic evaluation of altered genes following miR-152-3p manipulation

Cells (LNCaP: 400000 cells/well, and PC3: 150000 cells/well) were plated in 6-well, in the day before transfection. Cells were collected 72 h post transfection and RNA was extracted and used as template for cDNA synthesis. RT-qPCR was performed as previously described and data analyzed according to the comparative Ct method [17].

### Gene expression microarrays

RNA was extracted from tissue samples using TRIzol (Invitrogen by Life Technologies, Carlsbad, CA), as previously described [18], and 1 μg of RNA was processed into cDNA and hybridized to Affymetrix GeneChip Human Exon 1.0 ST arrays, following the manufacturer’s recommendations [19]. The Affymetrix Expression Console v1.1 software was used to obtain exon-level robust multi-array average (RMA)-normalized expression values for the core probe sets only. The data is freely available in GEO repository under accession number GSE42954.

### Luciferase assay

A reporter plasmid containing a binding site at NOL4 or TMEM97 3′UTR for miR-152-3p (GeneCopoeia, Rockville, MD, USA) was co-transfected into HEK293Ta cells using Lipofectamine 2000 transfection reagent (Invitrogen, Carlsbad, CA, USA). Thirty nanomolars of synthetic pre-miRNA was used. Luciferase activity was assessed with the Secrete-Pair™ Dual Luminescence Assay Kit (GeneCopoeia, Rockville, MD, USA) according to the manufacturer’s instructions. The ratio of luminescence intensities (RLU, Relative Light Unit) of the GLuc (Gaussia luciferase) over SEAP (secreted Alkaline Phosphatase) was obtained as follows: GLuc/SEAP, for each triplicate.

### Statistical analysis

Non-parametric tests (Kruskal-Wallis and Mann-Whitney *U* test) were used for group comparisons analysis for both expression and methylation levels for the two patient cohorts (IPO’s and TCGA) and for the in vitro assays. Correlations between expression levels and methylation were evaluated by Spearman’s correlation test. Data are shown as mean ± s.d., unless otherwise specified. Student’s *t* test was used for invasion assays. To evaluate the prognostic value of mir152 and *TMEM97* expression in PCa patients from the TCGA dataset, univariable (Log-rank test) and multivariable (Cox regression) analyses of disease-free survival were performed, where putative confounding effects (Gleason score and patients’ age) were considered. Disease-free survival was calculated from the date of the radical prostatectomy to the date of relapse, or date of last follow-up or death if relapse-free. For the purposes of survival analyses, high gene expression was considered for samples within the 90th percentile (10% of samples with highest gene expression values). All statistical tests were two-sided. All experiments were run in triplicate. Statistical analysis was carried out using Graph Pad Prism version 5 and IBM SPSS statistics version 22. Significance level was set at *p* < 0.05.

## Results

### Identification of hypermethylated and downregulated microRNAs in prostate cancer

**Fig. 1:**
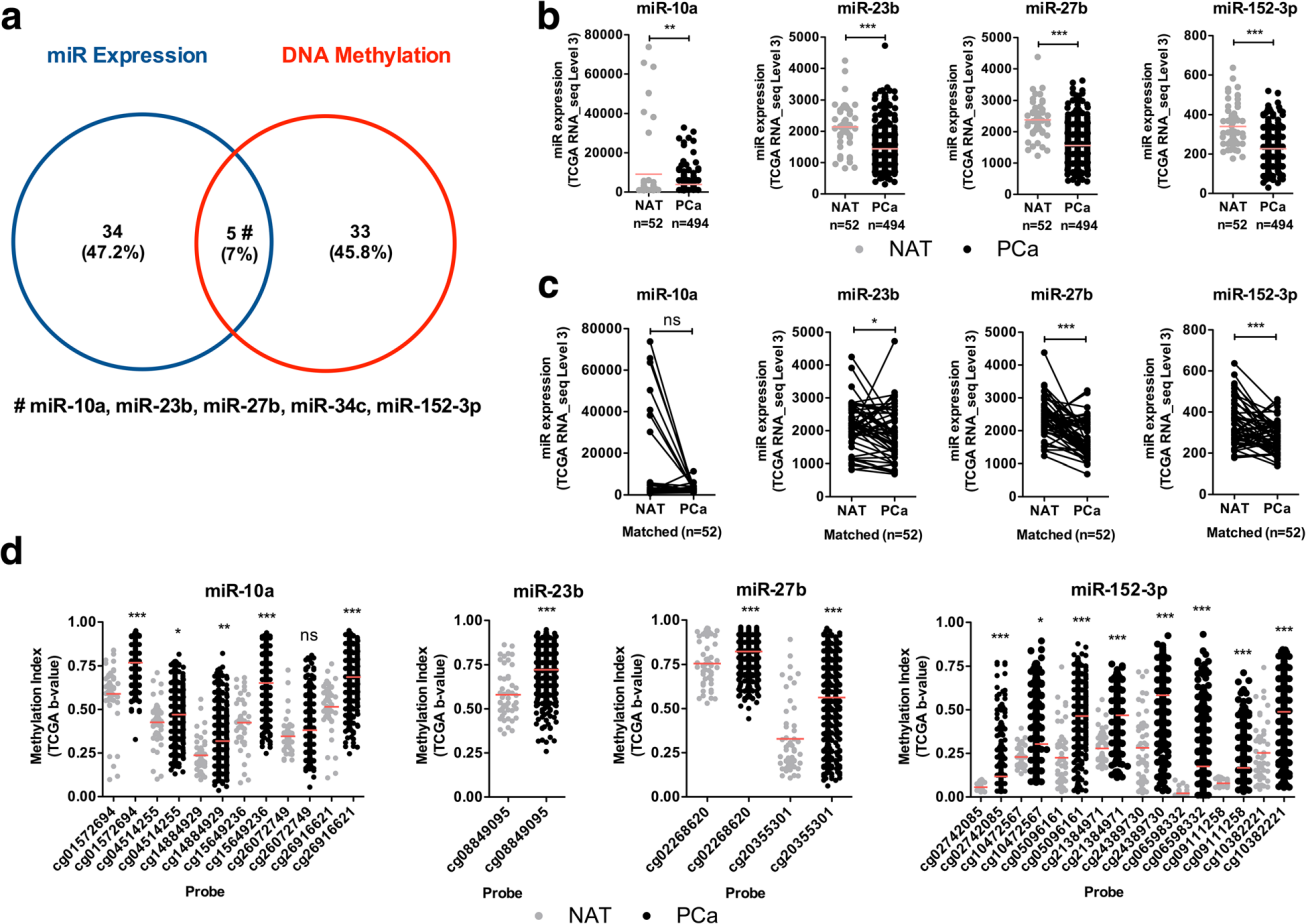
Identification of miRNAs downregulated by DNA methylation in prostate cancer, using a combinatorial approach. **a** Venn diagram of the intersection of the miR expression (Exiqon) versus DNA methylation (Infinium HumanMethylation450 BeadChip) for miRNA promoters. Intersection is shown for the downregulated miRNAs and hypermethylated miRNAs. The five common miRNAs based on expression level and DNA methylation in PCa tissues are miR-10a, miR-23b, miR-27b, miR-34c, and miR-152-3p. **b** Independent validation using the TCGA Prostate RNA-seq cohort for miR-10a, miR-23b, miR-27b, and miR-152-3p in PCa samples compared to NAT samples. **c** MiRNA expression analysis of 52 matched normal and PCa samples pairs using TCGA cohort. Except for miR-10a, all miRs were significantly downregulated in PCa. **d** DNA methylation levels (β-Values) for each probe in specific miRNA loci, comparing normal and PCa samples using TCGA Prostate 450 K cohort. Overall, DNA methylation gain (hypermethylation) was found in PCa samples. NAT: Normal Adjacent Tissue; PCa: Prostate Cancer; Mann-Whitney *U* test: *p < 0.05, **p < 0.01, ***p < 0.001

Global miRNA expression was assessed using microRNA Ready-to-Use PCR Human Panel (I + II) v2.0 (Exiqon, Vedbaek, Denmark). To identify differentially expressed miRNAs in PCa, compared to morphologically normal prostate tissue (MNPT), 740 miRNAs were profiled in 10 PCa tissues and 4 MNPT [20]. Using a cutoff value of log fold change < − 1.5, 39 miRs were found downregulated in PCa compared to MNPT (Additional file 1: Table S2 and Figure S1). From these miRNAs, 10 were selected for validation in a larger and independent dataset (miR-10a, miR-23b, miR-27b, miR-135b, miR-143, miR-152-3p, miR-187, miR-204, miR-205, miR-221), i.e., the miRNAseq expression data from PCa patients and matched normal samples deposited in The Cancer Genome Atlas (TCGA) (*n* = 326 and *n* = 52, respectively) (Fig. 1a–c and Additional file 1: Figure S2).

We also determined global differences in DNA methylation in the prostate tissues [21]. This cohort was composed of 5 MNPT and 25 PCa. DNA samples were hybridized on the Infinium DNA methylation BeadChip platform (Illumina), which analyzes more than 450,000 CpG sites in the genome [22]. After normalization, we filtered out poor-quality probes and those containing single nucleotide polymorphisms (SNPs; > 1%) [23] and copy number variations (CNV; > 5%) in the detection sequence. Following these filters, DNA methylation profiling disclosed 38 hypermethylated promoter regions in known miRNA regions, three of which were further validated in TCGA dataset (Additional file 1: Table S2 and Figure S3).

Because gene expression and DNA promoter methylation correlate with gene regulatory activity status, we merged the results of the two analytical platforms (expression by Exiqon platform and methylation profiling by Illumina) to identify miRNAs with decreased expression associated with promoter hypermethylation in PCa. From this combined analysis, 5 miRNAs emerged as simultaneously downregulated and hypermethylated in PCa: miR-10a, miR-23b, miR-27b, miR-34c, and miR-152-3p (Fig. 1a; Additional file 1: Table S2, highlighted in blue). In TCGA dataset, the common microRNAs were confirmed to be downregulated in primary PCa compared to noncancerous prostate tissues (Fig. 1b), but miR-34c showed no statistical significance. Moreover, and except for miR-10a, all displayed significantly lower expression in PCa in comparison with matched non-cancerous prostate tissues (Fig. 1c). Because miRNA’s promoter methylation status was available at TCGA database, these data were also retrieved and it confirmed our finding of increased methylation indexes in these miR’s obtained with the Infinium 450 K DNA methylation profiling platform (Fig. 1d). Then, we focused our study on miR-152-3p as it fulfilled the criteria for downregulation associated with promoter hypermethylation in PCa. Remarkably, we had already suggested that miR-152-3p was regulated by DNA methylation in PCa [21, 24], confirming previous published findings [25, 26] which support the observations that miR-152-3p is a common epigenetically regulated miRNA in PCa.

### MiR-152-3p expression and promoter methylation analysis in prostate cells

**Fig. 2:**
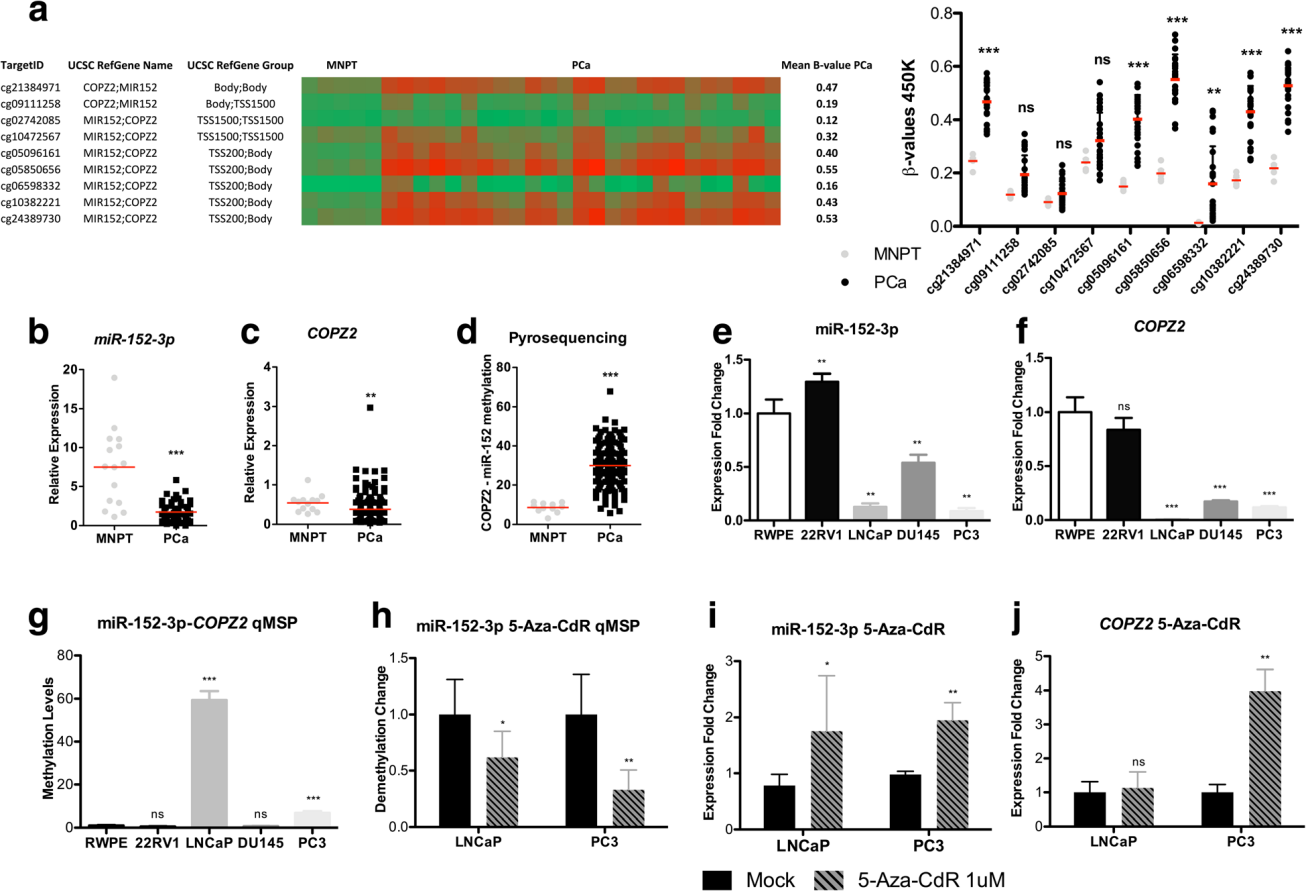
*COPZ2*-miR-152-3p transcriptional unit’s DNA methylation and expression validation in IPO Porto’s cohort of prostate samples. **a** HumanMethylation450 BeadChip data for miR-152-3p locus, showing a significant increase in cg21384971, cg05096161, cg05850656, cg06598332, cg10382221, and cg24389730. **b** Significant miR-152-3p downregulation in PCa (*n* = 100) compared to morphologically normal prostate tissues (MNPT, *n* = 14), as determined by RT-qPCR (*p* < 0.0001). **c** Significantly decreased *COPZ2* transcript levels in PCa samples (*p* = 0.0022). **d** *COPZ2*-miR-152-3p promoter hypermethylation in PCa samples determined by pyrosequencing (p = < 0.0001). **e** MiR-152-3p expression levels in PCa cell lines compared to benign RWPE cells [expression (E):1] assessed by RT-qPCR. Expression is significantly lower in LNCaP (E ~ 0.13), DU145 (E ~ 0.54) and PC3 (E ~ 0.09) cells. **f** Significantly lower *COPZ2* expression levels by RT-qPCR in LNCaP (E ~ 0.005), DU145 (E ~ 0.17) and PC3 (E ~ 0.12) cells compared to RWPE (E 1). **g** Prostate cell lines’ DNA methylation profiling. LNCaP (FC of methylation levels: ~ 59) and PC3 (FC: ~ 7) cells showed increased miR152-*COPZ2* promoter hypermethylation compared to RWPE (FC: 1), 22RV1 and DU145 cells. **h** A 72-h 5-Aza-CdR exposure, associated with significant decrease in promoter methylation levels of the transcriptional unit *COPZ2*-miR-152-3p in LNCaP (decreased ~ 40%) and PC3 (decreased ~ 68%) cells. LNCaP and PC3 cells miR-152-3p (**i**) and *COPZ2’s* (**j**) expression levels following 72-h exposure to 5-Aza-CdR associated with increased miR-152-3p expression levels (FC:1.75; 1.95, respectively) and *COPZ2* (FC:1.12; 3.98, respectively). Error bars represent the s.d. for three biological replicates. Mann-Whitney *U* test: *p < 0.05, **p < 0.01, ***p < 0.001. *β* values range from 0 to 1 and were determined by the ratio of the methylated probe intensity and the overall intensity (sum of methylated and unmethylated probe intensities). MNPT: Morphologically Normal Prostate Tissue; PCa: Prostate Cancer; 5-Aza-CdR: 5-aza-2-deoxycytidine

MiR-152-3p is located at chromosome 17q21.32, within an intronic region of *COPZ2*. Data from the 450 K array revealed that 9 probes targeting this locus were differentially methylated (Fig. 2a). The cg05850656 and cg24389730 showed the highest *β* values (0.55 and 0.53). These probes map to TSS200-Body for miR-152-3p-*COPZ2* locus. To further validate these findings, miR-152-3p (Fig. 2b) and *COPZ2* (Fig. 2c) expression levels were assessed in our cohort of prostatic tissues (PCa = 100 and MNPT = 14) and downregulation of both in PCa compared to MNPT was confirmed (*p* < 0.0001 and *p* = 0.0022, respectively).

In both patients’ cohorts, miR-152-3p and *COPZ2* expression levels were inversely and significantly correlated with methylation levels in PCa samples (*p* = − 0.444, *p* < 0.0001, and *p* = − 0.435, *p* < 0.0001 for IPO Porto cohort; *p* = − 0.331, *p* < 0.0001 and *p* = − 0.561, *p* < 0.0001, TCGA Cohort, respectively).

Pyrosequencing analysis demonstrated that the promoter shared by miR-152-3p and *COPZ2* was aberrantly methylated in PCa (Fig. 2d; *p* < 0.0001).

In LNCaP, DU 145, and PC3 cells, miR-152-3p and *COPZ2* expression levels were also significantly lower than those found on RWPE cells, which are benign epithelial prostate cells (Figs. 2e, f), whereas promoter methylation levels followed the opposite trend, specifically for the latter two cell lines—LNCaP and PC3 (Fig. 2g). These findings suggest that miR-152-3p is transcribed in parallel with its host gene, *COPZ2*. Exposure of PCa cells to demethylating agent 5-Aza-2-deoxycytidine (5-Aza-CdR) caused a 38 and 67% reduction in *COPZ2*-miR-152-3p promoter methylation levels, in LNCaP (*p* = 0.0411) and PC3 cells (*p* = 0.0043), respectively (Fig. 2h). Nonetheless, the impact in gene expression differed as miR-152-3p re-expression was observed in both cell lines (LNCaP, FC: 1.75; and PC3, FC: 1.94) (Fig. 2i), whereas *COPZ2* transcript levels were only significantly restored in PC3 cells (FC: 3.98) (Fig. 2j).

### MiR-152-3p attenuates malignant phenotype in vitro

**Fig. 3:**
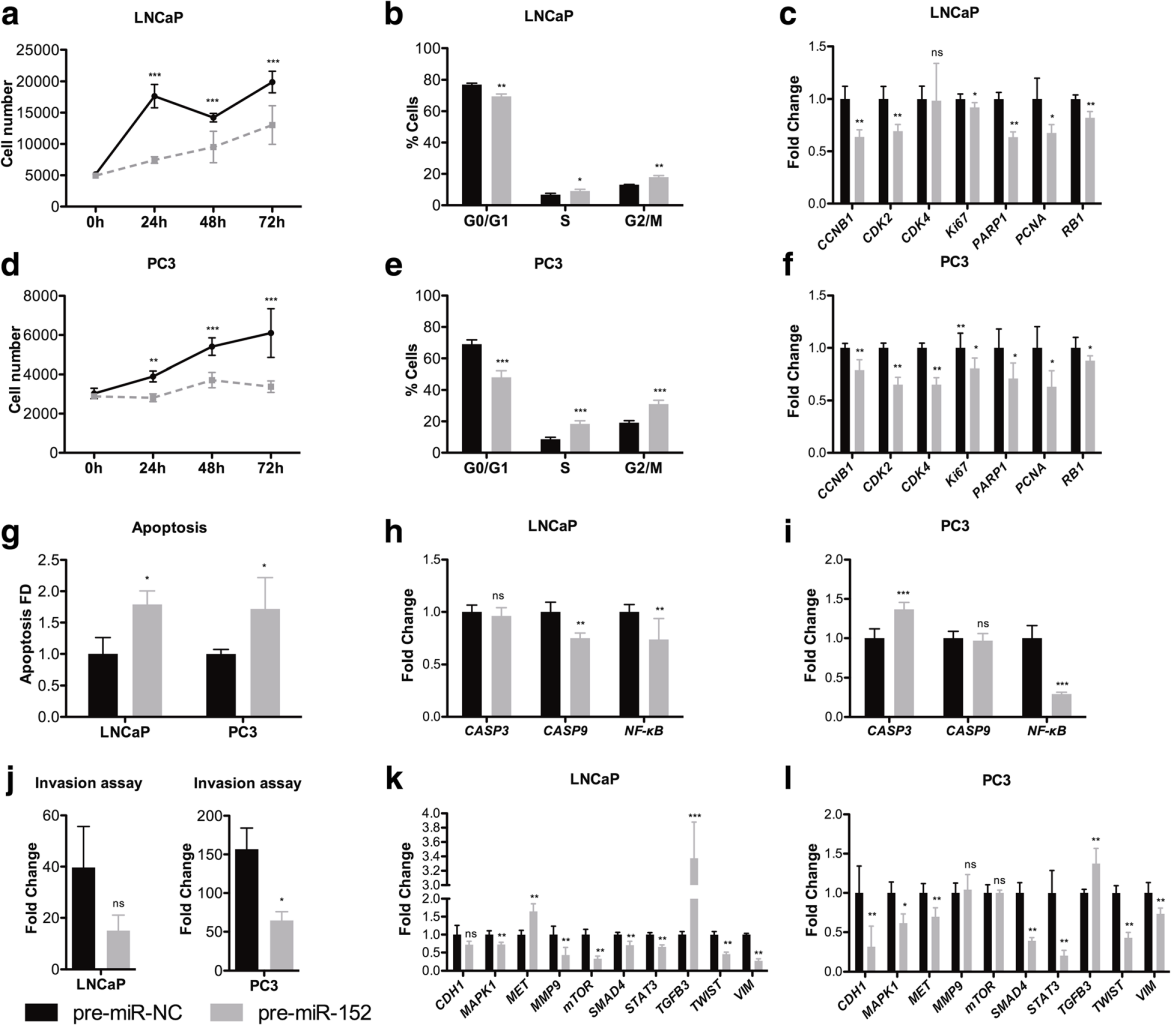
miR-152-3p overexpression associated with attenuated malignant features in LNCaP and PC3 cells. **a**, **d** miR-152-3p overexpression in LNCaP and PC3 cells significantly decreased cell viability compared to pre-miR-NC transfected cells (MTT assay at 24, 48, and 72 h). **b**, **e** Cell cycle arrest at S and G2/M phases was depicted for LNCaP and PC3 cells overexpressing miR-152-3p (cytometry analysis was performed 72 h after transfection). **c**, **f** Reduced transcription levels of several cell cycle-promoting genes in miR-152-3p overexpressing LNCaP and PC3 cells. **g** MiR-152-3p overexpression associated with significant increase in apoptosis compared to cells transfected with negative control miRNA. **h, i** Apoptosis-related genes’ expression levels were deregulated in PCa miR-152-3p overexpressing cells. Significantly reduced *NF-kB* levels were found in both miR-152-3p overexpressing cell lines. **j** MiR-152-3p forced expression in LNCaP and PC3 cells associated with a significant decrease number of cells invading through the Matrigel coated Boyden chamber assay. **k**, **l** Transcriptional deregulation of EMT and invasion-related genes in miR-152-3p transfected LNCaP and PC3. Differential *MET, mTOR and MMP9*S results suggest cell-specific gene regulation. *TGFB3* overexpression and EMT markers *STAT3*, *TWIST*, or *VIM* decrease was shared by both miR-152-3p overexpressing cells. Error bars represent the s.d. for three biological replicates. Mann-Whitney U-test: *p < 0.05, **p < 0.01, ***p < 0.001

Using in vitro assays, we found that miR-152-3p overexpression significantly decreased cell viability in both LNCaP and PC3 cells (Fig. 3a, d) and promoted a significant accumulation of cells in S and G2/M phases (Fig. 3b, e). Accordingly, at transcriptional level, both cell lines displayed a significant decrease of several cell cycle regulators (Fig. 3c, f). Conversely, miR-152-3p’s mimic transfection associated with increased apoptosis in LNCaP (*p* = 0.0286) and PC3 (*p* = 0.0286) cells (Fig. 3g; Additional file 1: Figure S4). These results were further supported by the significantly reduced NF-kB expression in both PCa cell lines, as well as significantly increased *CASP3* expression levels, although only in PC3 transfected cells (Fig. 3h, i). Moreover, miR-152-3p overexpression significantly reduced PC3 invasion ability in PC3 cells (*p* = 0.0286; Fig. 3j) and associated with specific epithelial–mesenchymal transition genes’ downregulation. Indeed, along with *TWIST* and *VIM* downregulation, *MAPK1*, *SMAD4*, and *STAT3* were significantly downregulated after miR-152-3p restored expression in both LNCaP and PC3 cells (Fig. 3k, l).

### miR-152-3p targets *NOL4* and *TMEM97*

**Fig. 4:**
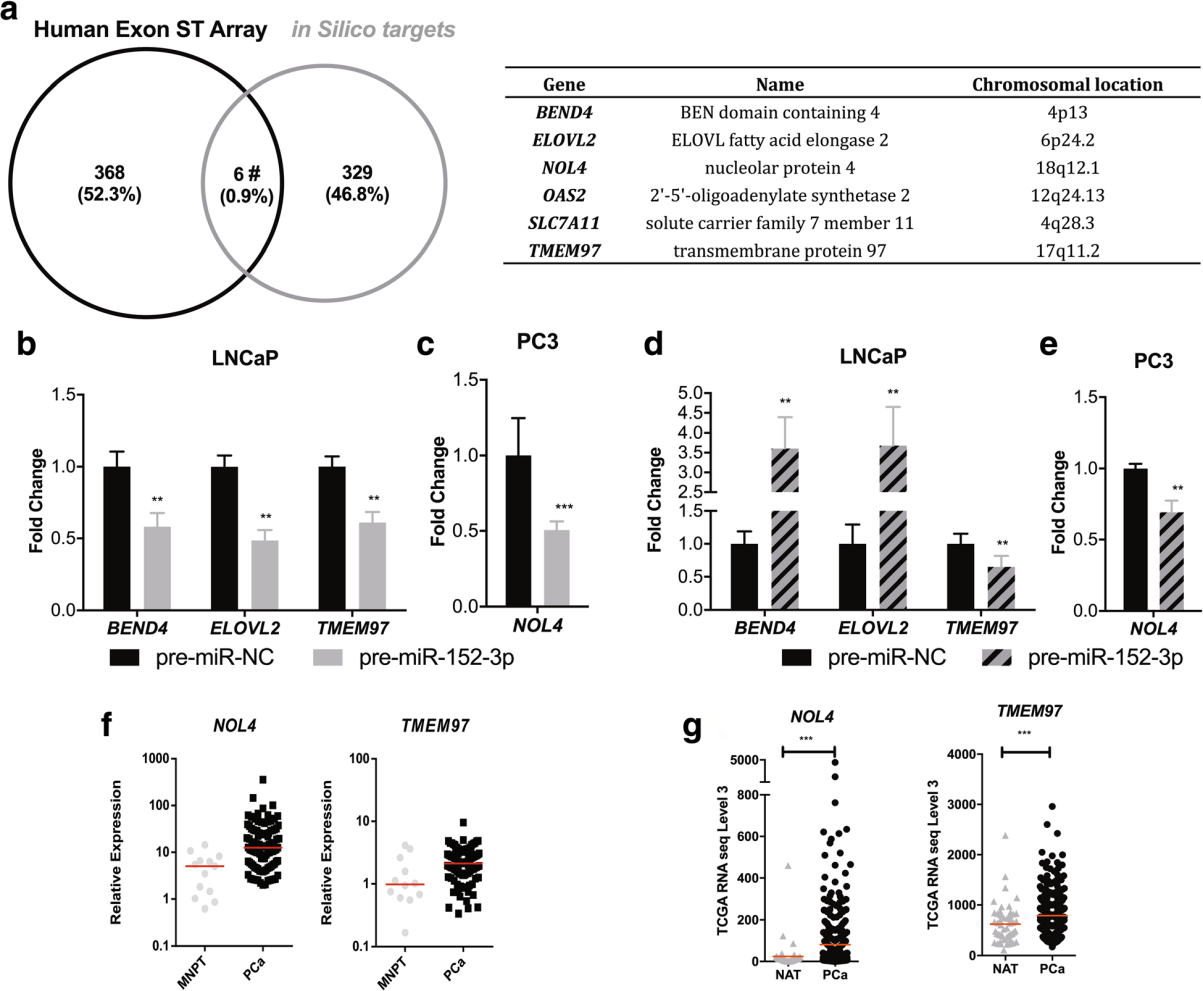
Identification of putative targets of miR-152-3p in PCa cell lines. **a** Genes selected for validation in our experimental settings: combining in silico prediction targets with genome-wide expression using GeneChip® Human Exon ST Array. **b** MiR-152-3p overexpression in LNCaP cells associated with significant decreased levels of *BEND4* (~ 40%)*, ELOVL2* (52%) and *TMEM97* (40%) as determined by RT-qPCR. **c** PC3 miR-152-3p’s transfected cells displayed significantly decreased *NOL4* expression (approximately 50%). **d**, **e** Effect of 5-Aza-CdR treatment in the selected target genes transcript levels in LNCaP revealed *TMEM97* downregulation (up to 35%), whereas in PC3 cells it associated with significantly decreased *NOL4* transcript levels (− 30%). *NOL4* and *TMEM97* upregulation in PCa tissues. **f** Significantly higher *NOL4* and *TMEM97* expression in PCa tissue samples (*n* = 100) compared with morphologically normal prostate tissue (*n* = 14), determined by RT-qPCR. **g** Expression levels in TCGA Prostate by RNA-seq cohort (NAT: *n* = 52; PCa: *n* = 497). MNPT: Morphologically Normal Prostate Tissue; NAT: Normal Adjacent Tissue; PCa: Prostate Cancer. Error bars represent the s.d. for three biological replicates. Mann-Whitney U-test: *p < 0.05, **p < 0.01, ***p < 0.001

Because the previous results suggested that miR-152-3p was an onco-suppressor, we sought to identify its targets for post-transcriptional regulation, using a combination of multiple in silico target prediction tools (putative targets must contain at least one miRNA response element (MRE)) (Additional file 1: Table S3) and a publicly available gene expression dataset. Among the 329 genes predicted as miR-152-3p targets in silico, only six—*BEND4*, *ELOVL2*, *NOL4*, *OAS2*, *SLC7A11*, and *TMEM97*—disclosed a gene expression log fold change > 1.5 in PCa samples (*n* = 368) analyzed by GeneChip Human Exon ST Array (Fig. 4a). Moreover, forced expression of miR-152-3p caused a significant downregulation of *BEND4*, *ELOVL2*, and *TMEM97* in LNCaP cells (Fig. 4b), whereas in PC3 cells, only *NOL4* transcript levels significantly diminished following miR-152-3p’s overexpression (Fig. 4c). LNCaP cells overexpressing miR-152-3p exposed to 5-Aza-CdR showed significantly reduced *TMEM97* expression levels (Fig. 4d), and *NOL4* was downregulated in PC3 cells (Fig. 4e).

To further confirm the biological significance of our previous findings, *NOL4* and *TMEM97* expression levels were assessed in two independent cohorts of PCa patients (from our institution and from TCGA). Remarkably, we found that *TMEM97* and *NOL4* expression levels were significantly upregulated in PCa cases from both cohorts, compared to normal prostate tissues (*p* = 0.0132 and *p* = 0.0004, respectively, in IPO Porto’s cohort; *p* < 0.0001 and *p* < 0.0001, respectively, in TCGA cohort; Fig. 4f, g).

### *TMEM97* knockdown decreases cell proliferation

**Fig. 5:**
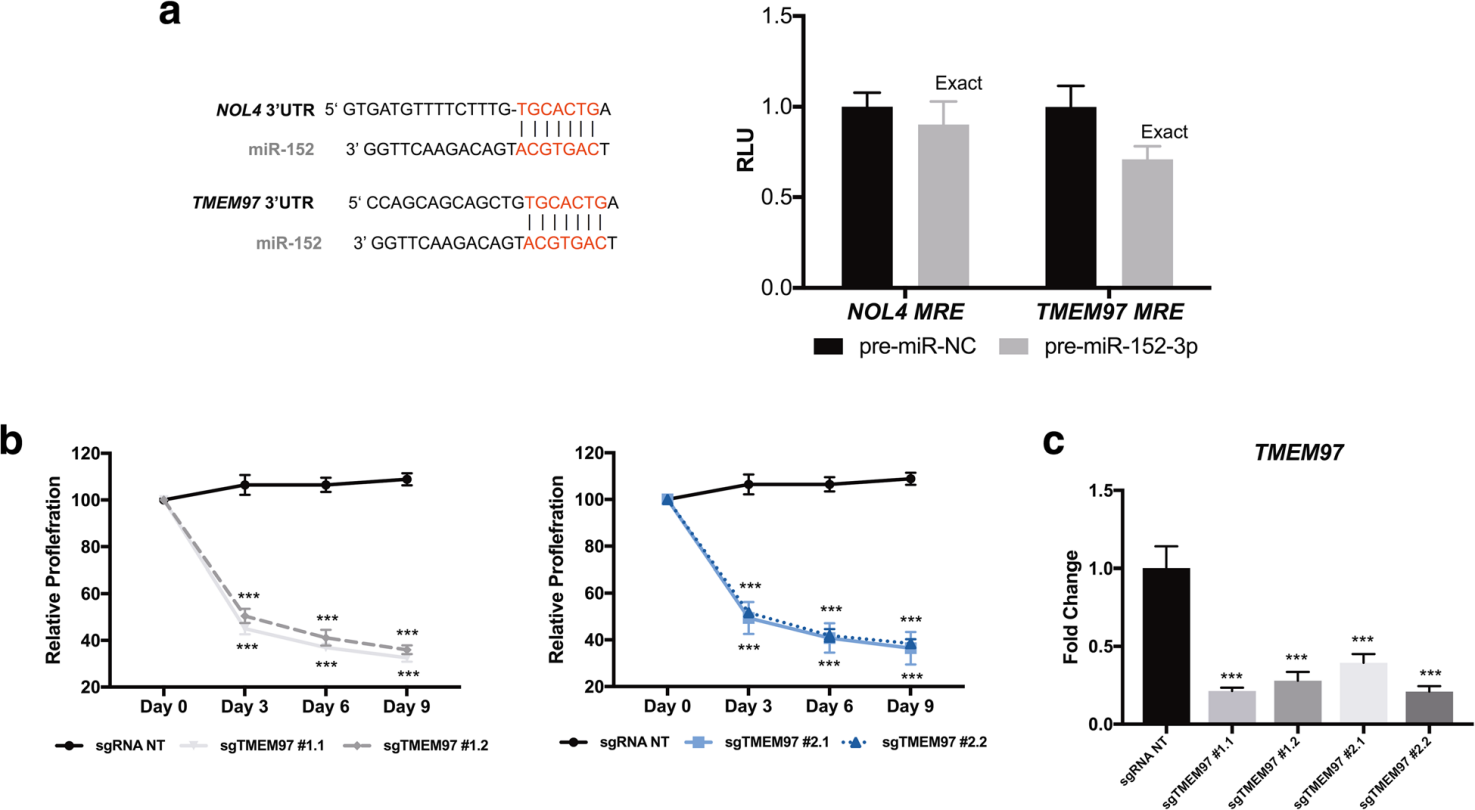
*NOL4* and *TMEM97* are miR-152-3p targets **a** Schematic representation of the miR-152-3p’s MRE in *NOL4* and *TMEM97*. Luciferase activity in HEK293Ta cells co-transfected with reporter constructs containing *NOL4* MRE or *TMEM97* MRE and either pre-miR-152-3p or pre-miR-NC. **b** Validation of candidate sgRNAs for *TMEM97* by a competitive proliferation assay in LNCaP cells. **c** SgRNA NT indicates a nontargeting. GFP levels were measured by flow cytometry. Values are normalized to day 0 (*T* = 0). 3′UTR: 3′ Untranslated Region; MRE: miRNA response element Mann-Whitney *U* test: *p < 0.05, **p < 0.01, ***p < 0.001

Since both *NOL4* and *TMEM97* 3′UTRs contain a MRE for miR-152-3p (Fig. 5a, left panel), the functional interaction between miR-152-3p and *NOL4*, on the one hand, and *miR-152-3p* and *TMEM97*, on the other hand, was investigated using luciferase assays. Interestingly, a 30% reduction (*p* = 0.0022) in luciferase activity for the *TMEM97* MRE was found, although only a 10% decrease was depicted for the *NOL4* MRE (Fig. 5a, right panel).

Thus, we tested 4 individual sgRNAs designed to target *TMEM97.* We validated the candidate sgRNAs with a competitive proliferation assay. LNCaP cells were transduced with the indicated sgRNAs and allowed to proliferate for 9 days. No clonal selection was performed; thus, cells’ population not only comprises a pool of homozygous/heterozygous mutants, but also unmutated cells (Additional file 1: Figure S5).

CRISPR-Cas9-mediated knockout of *TMEM97* with four different sgRNAs (sgTMEM97 exon#1 and exon#2) in LNCaP cells resulted in a significant decrease in proliferation (Fig. 5b). Remarkably, all four sgRNAs significantly diminished *TMEM97* mRNA levels (Fig. 5c), probably due to nonsense-mediated decay [27]. Cas9-nuclease activity generates DNA double-strand breaks that result in deletions and insertions at the vicinity of the sgRNA recognition site. We examined the range of deletions/insertions caused by each sgRNA targeting *TMEM97* (Additional file 1: Figure S5)*.* All the sgTMEM97 caused on-target indels, with a percentage ranging from 42.3–79.2%.

Overall, the sgRNAs targeting *TMEM97* mimicked the restoration of miR-152, suggesting that this gene is required for LNCaP cells growth.

### miR-152, *TMEM97*, and *NOL4* clinical potential in PCa

**Fig. 6:**
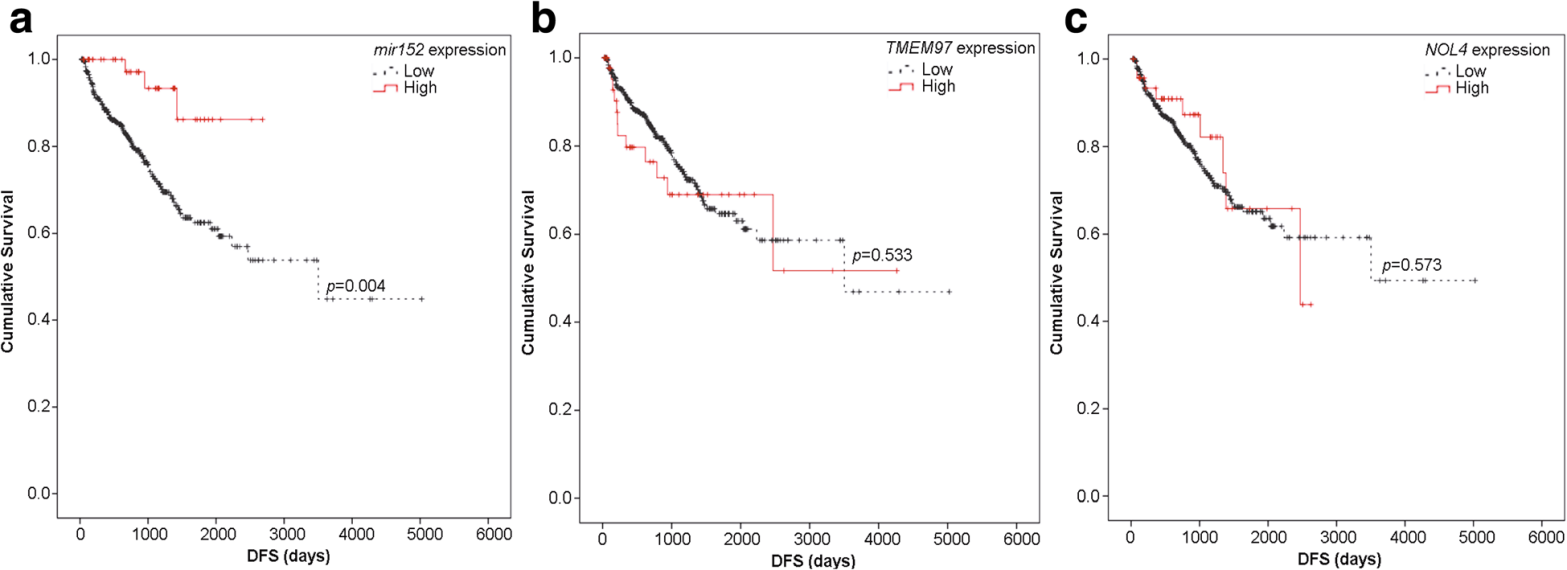
miR-152-3p is valuable prognostic biomarker in PCa. **a**–**c** Disease-free survival analyses were performed in PCa patients from the TCGA dataset. **a** High miR-152-3p expression levels significantly associated with longer disease-free survival (*p* = 0.004; Log-rank test). **b** *TMEM97* and **c** *NOL4* expression do not present prognostic value

Lastly, to further evaluate the biological significance of the previous findings in primary PCa, the prognostic value of miR-152, *NOL4*, and *TMEM97* expression levels was assessed in both PCa patients’ cohorts. Although, no associations were found in IPO Porto’s cohort, in TCGA’s cohort, higher miR-152-3p expression levels (90th percentile) predicted longer disease-free survival in univariable analysis (Log-rank test *p* = 0.004; Fig. 6a) and in multivariable Cox regression analysis adjusted for patients’ age and Gleason score (HR = 0.228, *p* = 0.012; Table 2). However, *TMEM97* or *NOL4* expression levels did not significantly associate with PCa patients’ outcome (Fig. 6b, c, Table 2).

**Table 2:** Disease-free survival analysis by Cox regression in TCGA patients

DFS	Multivariable analysis		
	HR	95% CI for HR	*p* value
mir152			
mir152 expression level^a^	*0.228*	*0.072–0.722*	*0.012*
Age	0.999	0.970–1.028	0.928
Gleason Score^b^			
6			*< 0.0001*
7	5.528	0.754–40.536	0.093
8	*11.104*	*1.455–84.709*	*0.020*
9	*23.559*	*3.247–170.946*	*0.002*
10	*24.146*	*1.502–388.242*	*0.025*
*TMEM97*			
*TMEM97* expression level^a^	1.007	0.550–1844	0.980
Age	1.000	0.971–1.029	0.978
Gleason Score^b^			
6			*< 0.0001*
7	5.369	0.732–39.373	*0.098*
8	*10.978*	*1.438–83.790*	*0.002*
9	*23.087*	*3.179–167.641*	*0.005*
10	30.812	2.786–340.785	0.978
*NOL4*			
*NOL4* expression level^a^	0.778	0.392–1.545	0.473
Age	1.000	0.972–1.029	0.979
Gleason Score^b^			
6			*< 0.0001*
7	5.309	0.724–38.935	0.101
8	*10.967*	*1.438–83.629*	*0.021*
9	*22.911*	*3.158–166.213*	*0.002*
10	*31.102*	*2.811–344.117*	*0.005*

*p* value significant when *p* < 0.05

*HR* hazard ratio, *CI* confidence interval.

^a^Reference group: high expression level

^b^Reference group: Gleason Score 6

## Discussion

MiRNAs are key players in cellular differentiation and homeostasis, being involved in regulation of transcriptional programs through the elimination of aberrant transcripts and suppression of random fluctuations in transcript copy number [28]. Thus, its deregulation impairs cellular homeostasis and is involved in the emergence of several pathologies, including PCa.

In this study, we aimed to extend current knowledge on the impact of epigenetic deregulation of miRNAs expression in PCa. For that purpose, we used a combined analysis that allowed the identification of downregulated and aberrantly methylated microRNAs. Interestingly, only 8% of downregulated miRNAs in PCa tissues were found to be simultaneously aberrantly methylated. Thus, promoter hypermethylation does not seem to be a prevalent mechanism underlying microRNA downregulation in this cancer model, and other causes, whether genetic [29], epigenetic [30] or microenvironment-related (e.g., abnormal AR signaling [31]), are likely to be more frequent. Notwithstanding, we have recently shown that aberrant microRNA promoter methylation might constitute a clinically useful tool for PCa detection and prognostication [24]. Although the number of candidate microRNAs was small, the combined approach used in this study seems to be more robust and efficient than each strategy (i.e., micro-RNA expression analysis and differential methylation mapping) alone, considering the significantly higher proportion of validated candidates obtained compared to previous studies from our group [15, 21]. Moreover, the results also validate this approach as it confirmed previous reports on miR-23b and miR-27b (members of the cluster miR-23b/27b/24-1) downregulation associated with promoter methylation in PCa [32–34]. Remarkably, two novel microRNAs within this category were found—miR-10a and miR-152-3p—although only the latter was validated in two independent datasets.

Interestingly, several miRNAs, including miR-135b, miR-143, miR-187, miR-204, miR-205 and miR-221 that were commonly downregulated in the Exiqon expression dataset, were also downregulated in TCGA dataset. In contrast, the number of putatively downregulated microRNAs due to aberrant DNA methylation found in the HumanMethylation450 BeadChip and validated in TCGA dataset was much smaller. Thus, our data indicate that miRNA expression’s profiling is more likely to identify bona fide miRNA deregulated due to promoter methylation compared to DNA methylation profiling, as aberrant DNA methylation might not indicate effective transcriptional silencing. Nevertheless, DNA methylation profiling might be particularly suitable for integrative analytic approaches [35].

Because miR-152-3p fulfilled the criteria for methylation-associated downregulation and it had not been previously reported in PCa, we sought to investigate its role in prostate carcinogenesis. Our study indicates that miR-152-3p is a sense-oriented intronic miRNA that forms a transcriptional unit (TU) with the respective host gene, *COPZ2*, being processed as part of the host gene mRNA [36, 37]. Globally, aberrant promoter methylation associated with simultaneous downregulation of *COPZ2*-miR152 expression. This further supports that host gene promoter methylation status is, indeed, associated with miRNAs deregulation, as previously suggested [26]. Nonetheless, the results of 5-Aza-CdR exposure in LNCaP suggest that this is not the only mechanism regulating *COPZ2*-miR152 expression, and we are tempted to speculate whether an independent promoter might be located upstream in the host gene, as reported for other genes [38, 39].

MiR-152-3p promoter methylation has also been reported in endometrial cancer [40] and in MLL-rearranged infant acute lymphoblastic leukemia [41]. In accordance with previous reports, our results also suggest an onco-suppressor function for miR-152-3p in PCa. Indeed, in non-small cell lung cancer miR-152-3p suppressed cell proliferation, colony formation, migration and invasion [42], in endometrial cancer miR-152-3p restored expression prevented tumor cell growth both in vitro and in vivo [40], and in ovarian cancer miR-152-3p was suggested to contribute to cisplatin resistance in vitro and in vivo through direct *DNMT1* targeting [43]. Moreover, in the TCGA PCa cohort, *DNMT1* upregulation associated with miR-152 promoter hypermethylation (Pearson’s = 0.12, *p* = 0.0236), although with limited statistical significance [26]. Interestingly, in prostatectomy samples, lower miR-152-3p expression levels were previously significantly associated with higher risk for biochemical recurrence, although only in univariable analysis [44]. Concordantly, our study shows miR-152-3p has prognostic value, although only in the TCGA cohort. Daniunaite et al. showed*,* using Kaplan-Meier analysis, that mir-152 promoter hypermethylation had a significant negative impact on BCR-free survival [26]. However, in a recent study aiming at PCa early detection, miR-152-3p expression did not discriminate PCa patients from the matched healthy controls in plasma samples [45]. Moreover, *TMEM97* and *NOL4* expression levels did not associate with patients’ prognosis in both cohorts.

In vitro functional assays demonstrated that miR-152-3p controls cell viability in PCa cells acting as S and G2/M cell-cycle transitions regulator, in both cell lines tested, but more expressively PC3 cells. However, these effects might be cell-context dependent. In line with functional assays’ results, miR-152-3p overexpression associated with different specific transcript alterations depending on the tested cell line. Our results indicate that miR-152-3p fine-tunes the expression of several genes involved in the MAPK/ERK, TFG-Beta, JAK–STAT3 and EMT pathways. Thus, our data not only supports that miR-152-3p is a promising molecular target that inhibits PCa cell cycle and invasion [25, 46], but also demonstrates its role in apoptosis regulation. Since these biological processes are critical for cancer progression, a major role for miR-152-3p in PCa progression might be anticipated.

To better understand the role of miR-152-3p, we attempted to identify putative targets. In silico analysis followed by validation in two independent patient cohorts indicated *NOL4* (nucleolar protein 4) and *TMEM97* (transmembrane protein 97) as miR-152-3p targets. Although no information is available for the role of *NOL4* in cancer, *TMEM97* has been shown to be upregulated in several malignancies, including glioma [47] as well as colorectal [48] and ovarian [49] cancers. Interestingly, in glioma cells, *TMEM97* depletion inhibited cancer cell growth and metastasis formation, in parallel with deregulation of EMT-related genes. In our model, *TMEM97* disruption decreased LNCaP proliferation, further suggesting its involvement in cell growth. Remarkably, increased *TMEM97* expression correlated with shorter survival in glioma [47], ovarian [49], non-small cell lung [50], and colorectal [48] cancer patients. Moreover, *TMEM97* cytoplasmic expression has been positively correlated with *PCNA* expression [48], which acts as a scaffold to recruit proteins involved in DNA replication or DNA repair, being required for post-replication repair [51]. Remarkably, in our study, miR-152-3p overexpression associated with *PCNA* downregulation in both cell lines. Therefore, since *TMEM97* might be functionally associated with *PCNA* [52], it might suggest that both are controlled by miR-152-3p. Concerning other previously reported miR-152-3p’s targets, we were not able to confirm DNMT1 as a miR-152-3p’s direct target [25, 43, 53].

## Conclusions

In conclusion, this study uncovered novel miRNAs downregulated by aberrant DNA methylation, including a transcriptional unit formed by *COPZ2*-miR-152-3p. Furthermore, it was demonstrated that miR-152-3p downregulation is a common feature in PCa, favoring the acquisition and development of the malignant traits, as in vitro miR-152-3p’s restored expression attenuated PCa cell phenotype, by impairment of cell viability, cell cycle progression and invasion, through targeting of several genes involved in critical cancer-related pathways.

## Additional file

Additional file 1: Supplementary information. Table S1 Primers and TaqMan assays used in this study; Table S2 microRNAs selected for evaluation in TCGA dataset; Table S3 Putative miR-152 target genes determined by multiple in silico prediction tools; Figure S1 Flow chart depicting the different steps followed to ascertain downregulated miRNAs in PCa; Figure S2 Representative examples of downregulated microRNAs at the TCGA cohort from the initial microRNA profiling by Exiqon; Figure S3 Representative examples of microRNAs downregulated at the TCGA cohort derived from the DNA methylation mapping by 450 K Illumina’s array; Figure S4 Efficiency of miR-152 mimic’s transfection in PCa cell lines; Figure S5 TIDE analysis of deletions/insertions caused by each sgRNA targeting TMEM97. (PDF 4188 kb)

## Abbreviations

5-Aza-CdR: 5-Aza-2-deoxycytidine
AGO: Argonaute
CNV: Copy Number Variations
FFPE: Formal-Fixed and Paraffin-Embedded
MAF: Minor Allele Frequency
miRNAs: microRNAs
MNPT: Morphologically Normal Prostate Tissue
NAT: Normal Adjacent Tissue
*NOL4*: Nucleolar Protein 4
PCa: Prostate Cancer
qMSP: quantitative Methylation Specific PCR
SNP: Single Nucleotide Polymorphisms
*TMEM97*: Transmembrane Protein 7
