# Supplementary material for: A multiplatform approach identifies miR-152-3p as a common epigenetically regulated onco-suppressor in prostate cancer targeting TMEM97
Source: Clin Epigenetics. 2018 Mar 27;10:40. doi: 10.1186/s13148-018-0475-2 (PMC5870254; doi:10.1186/s13148-018-0475-2)
Supplement: Supplementary file 1 — Supplementary information. Table S1 Primers and TaqMan assays used in this study; Table S2 microRNAs selected for evaluation in TCGA dataset; Table S3 Putative miR-152 target genes determined by multiple in silico prediction tools; Figure S1 Flow chart depicting the different steps followed to ascertain downregulated miRNAs in PCa; Figure S2 Representative examples of downregulated microRNAs at the TCGA cohort from the initial microRNA profiling by Exiqon; Figure S3 Representative examples of microRNAs downregulated at the TCGA cohort derived from the DNA methylation mapping by 450 K Illumina’s array; Figure S4 Efficiency of miR-152 mimic’s transfection in PCa cell lines; Figure S5 TIDE analysis of deletions/insertions caused by each sgRNA targeting TMEM97. (PDF 4188 kb) [file 13148_2018_475_MOESM1_ESM.pdf]

Supplementary Information

Ramalho-Carvalho *et* al:

Table S1 – Primers and TaqMan assays used in this study

Table S2 – microRNAs selected for evaluation in TCGA dataset.

Table S3 –Putative miR-152 target genes determined by multiple *in silico* prediction tools.

Supplementary Figures Legends:

Figure S1 - Flow chart depicting the different steps followed to ascertain downregulated miRNAs in PCa.

Figure S2 - Representative examples of downregulated microRNAs at the TCGA cohort from the initial microRNA profiling by Exiqon.

Figure S3 - Representative examples of microRNAs downregulated at the TCGA cohort derived from the DNA methylation mapping by 450K Illumina's array.

Figure S4 - Efficiency of miR-152 mimic's transfection in PCa cell lines

Figure S5 – TIDE analysis of deletions/insertions caused by each sgRNA targeting TMEM97.

Table S1 – Primers and TaqMan assays used in this study

	5'- Sequence – 3'	Figure
miR152-pyro_F	GGGTTTAAGTTTTGTTATGTATTGATTGT	2
miR152-pyro_R	[Btn]AAATCCAACCCRACCAAAAATCAACTA	2
miR152-pyro_S	GAGTYGGAGTGTATTATAGAATT	2
ACTBq_MSP_F	TGGTGATGGAGGAGGTTTAGTAAGT	2
ACTBq_MSP _R	AACCAATAAAACCTACTCCTCCCTTAA	2
miR-152_qMSP_F	TCGTCGTTCGGGATTTTC	2
miR-152_qMSP_R	ACTAACCACGTCCGCACC	2
BGUS_qPCR_F	CTCATTTGGAATTTTGCCGATT	2,3,4,5
BGUS_qPCR_R	CCGAGTGAAGATCCCCTTTTTA	2,3,4,5
COPZ2_qPCR_F	AATGTCTTCAACAAGACCAG	2
COPZ2_qPCR_R	ATAGGAAGAGGTCAATGCT	2
CCNB1_qPCR_F	GCCTGAGCCTATTTTGGTTGA	3
CCNB1_qPCR_R	CTTCTTCTGCAGGGGCACAT	3
CDH1_qPCR_F	CTTTGACGCCGAGAGCTACA	3
CDH1_qPCR_R	AAATTCACTCTGCCCAGGACG	3
CDK4_qPCR_F	GCCTCGAGATGTATCCCTGC	3
CDK4_qPCR_R	GTCAGCATTTCCAGCAGCAG	3
MAPK1_qPCR_F	GAAGCATTATCTTGACCAGC	3
MAPK1_qPCR_R	TCCATGGCACCTTATTTTTG	3
MMP9_qPCR_F	TTCAGGGAGACGCCCATTTC	3
MMP9_qPCR_R	AACCGAGTTGGAACCACGAC	3
NF-kB_qPCR_F	GCTTAGGAGGGAGAGCCCA	3
NF-kB_qPCR_R	CTGCCATTCTGAAGCCGGG	3
PCNA_qPCR_F	GCTCTTCCCTTACGCAAGTCT	3
PCNA_qPCR_R	AGTCTAGCTGGTTTCGGCTT	3
SMAD4_qPCR_F	CCAGCTCTGTTAGCCCCATC	3
SMAD4_qPCR_R	TACTGGCAGGCTGACTTGTG	3
STAT3_qPCR_F	GAAACAGTTGGGACCCCTGA	3
STAT3_qPCR_R	GCTCCATTGGGAAGCTGTCA	3
TWIST_qPCR_F	TTCTCGGTCTGGAGGATGGA	3
TWIST_qPCR_R	TTCTCTGGAAACAATGACATCTAGG	3
BEND4_qPCR_F	TCGTTTCGGAAGCCGGAC	4
BEND4_qPCR_R	CCTCGCCAATGCCTGGAG	4

ELOVL2_qPCR_F	GCTGCGGATCATGGAACATC	4
ELOVL2_qPRC_R	ACCACCCTCTGACTCGAGAA	4
NOL4_qPCR_F	CCTGTCAAGACCACGGATGG	4,5
NOL4_qPCR_R	TCTTCAACCACAGCTACCCG	4,5
TMEM97_qPCR_F	AGTCGAGTTTAGAAACCTGCTGA	4,5
TMEM97_qPCR_R	GACTTAAACCAGGCTGGGGG	4,5
sgTMEM97_Exon1.1_R	aaacCCATAGTCTGTCGGTTGGGCc	5
sgTMEM97_Exon1.1_F	CACCgGCCCAACCGACAGACTATGG	5
sgTMEM97_Exon1.2_F	CACCGCCGGAGCCCCCATAGTCTGT	5
sgTMEM97_Exon1.2_R	aaacACAGACTATGGGGGCTCCGGC	5
sgTMEM97_Exon2.1_R	aaacGCTGAAGTGGTATGCTAAGGC	5
sgTMEM97_Exon2.1_F	CACCGCCTTAGCATACCACTTCAGC	5
sgTMEM97_Exon2.2_R	aaacTAGCATACCACTTCAGCAGGC	5
sgTMEM97_Exon2.2_F	CACCGCCTGCTGAAGTGGTATGCTA	5
TMEM97_sanger_exon1_F	CGCTCACAACCGCAATCTACA	5
TMEM97_sanger_exon1_R	GAACCCAGAGTGCGCGAG	5
TMEM97_sanger_exon2_F	GTGCCTACCAAGTGCCAGAT	5
TMEM97_sanger_exon2_R	GACCATCCCCTTCCCTTTGG	5
**Primer**	**Reference**	**Figure**
GUSB	Hs99999908_m1	3
CASP3	Hs00234387_m1	3
CASP9	Hs00609640_m1	3
CDK2	Hs01548894_m1	3
KI67	Hs01032437_m1	3
PARP1	Hs00242302_m1	3
RB1	Hs01078066_m1	3
MET	Hs01565584_m1	3
mTOR	Hs_00234508_m1	3
TGFB3	Hs01086000_m1	3
VIM	Hs00185584_m1	3

Table S2 – microRNAs selected for evaluation in TCGA dataset.

miRNA expression profile - Exiqon

MIRNA	Fold Change	Gene name	MiRBase ID	Family	5p accession	3p accession	Chromosome	Start	End	Strand	origin (gene) ▽	Node of origin (family) ▽
hsa-miR-187	-3,1	Hsa-Mir-187	hsa-mir-187	MIR-187	MIMAT0004561	MIMAT0000262	chr18	35904835	35904892	-	Osteichthyes	Osteichthyes
hsa-miR-520d-3p	-2,9	Hsa-Mir-430-P34	hsa-mir-520d	MIR-430	MIMAT0002855	MIMAT0002856	chr19	53720111	53720169	+	Catarrhini	Vertebrata
hsa-miR-551b	-2,8	Hsa-Mir-551-P2	hsa-mir-551b	MIR-551	MIMAT0004794	MIMAT0003233	chr3	168551875	168551934	+	Vertebrata	Vertebrata
hsa-miR-1270	-2,8	Hsa-Mir-1270	hsa-mir-1270	MIR-1270	MIMAT0005924	None	chr19	20399284	20399342	-	H. sapiens	H. sapiens
hsa-miR-224	-2,7	Hsa-Mir-224	hsa-mir-224	MIR-224	MIMAT0000281	MIMAT0009198	chrX	151958583	151958651	-	Eutheria	Eutheria
hsa-miR-34c-5p	-2,7	Hsa-Mir-34-P2b	hsa-mir-34c	MIR-34	MIMAT0000686	MIMAT0004677	chr11	111513451	111513505	+	Vertebrata	Bilateria
hsa-miR-296-3p	-2,5	Hsa-Mir-296	hsa-mir-296	MIR-296	MIMAT0000690	MIMAT0004679	chr20	58817626	58817681	-	Eutheria	Eutheria
hsa-miR-302c	-2,4	Hsa-Mir-430-P3	hsa-mir-302c	MIR-430	MIMAT0000716	MIMAT0000717	chr4	112648368	112648423	-	Tetrapoda	Vertebrata
hsa-miR-383	-2,4	Hsa-Mir-383	hsa-mir-383	MIR-383	MIMAT0000738	MIMAT0026485	chr8	14853443	14853504	-	Tetrapoda	Tetrapoda
hsa-miR-424	-2,2	Hsa-Mir-15-P1d	hsa-mir-424	MIR-15	MIMAT0001341	MIMAT0004749	chrX	134546643	134546701	-	Eutheria	Olfactores
hsa-miR-1911	-2,2	Hsa-Mir-1911	hsa-mir-1911	MIR-1911	MIMAT0007885	MIMAT0007886	chrX	114763195	114763253	+	Boreoeutheria	Boreoeutheria
hsa-miR-509-3-5p	-2,1	Hsa-Mir-506-P4c	hsa-mir-509-3	MIR-506	MIMAT0004975	MIMAT0002881	chrX	147259661	147259717	-	H. sapiens	Eutheria
hsa-miR-483-3p	-2,1	Hsa-Mir-483	hsa-mir-483	MIR-483	MIMAT0004761	MIMAT0002173	chr11	2134140	2134202	-	Eutheria	Eutheria
hsa-miR-205	-2,1	Hsa-Mir-205-P1	hsa-mir-205	MIR-205	MIMAT0000266	MIMAT0009197	chr1	209432166	209432224	+	Vertebrata	Vertebrata
hsa-miR-135b	-2,1	Hsa-Mir-135-P3	hsa-mir-135b	MIR-135	MIMAT0000758	MIMAT0004698	chr1	205448323	205448383	-	Vertebrata	Chordata
hsa-miR-525-3p	-2,0	Hsa-Mir-430-P44	hsa-mir-525	MIR-430	MIMAT0002838	MIMAT0002839	chr19	53697547	53697605	+	Catarrhini	Vertebrata
hsa-miR-23b	-1,9	Hsa-Mir-23-P2	hsa-mir-23b	MIR-23	MIMAT0004587	MIMAT0000418	chr9	95085227	95085288	+	Vertebrata	Vertebrata
hsa-miR-152	-1,9	Hsa-Mir-148-P3	hsa-mir-152	MIR-148	MIMAT0026479	MIMAT0000438	chr17	48037174	48037232	-	Vertebrata	Vertebrata
hsa-miR-204	-1,8	Hsa-Mir-204-P1	hsa-mir-204	MIR-204	MIMAT0000265	MIMAT0022693	chr9	70809993	70810052	-	Vertebrata	Vertebrata
hsa-miR-519d	-1,8	Hsa-Mir-430-P29	hsa-mir-519d	MIR-430	MIMAT0026610	MIMAT0002853	chr19	53713361	53713422	+	Catarrhini	Vertebrata
hsa-miR-146b-3p	-1,8	Hsa-Mir-146-P2	hsa-mir-146b	MIR-146	MIMAT0002809	MIMAT0004766	chr10	102436520	102436578	+	Vertebrata	Vertebrata
hsa-miR-628-3p	-1,8	Hsa-Mir-628	hsa-mir-628	MIR-628	MIMAT0004809	MIMAT0003297	chr15	55372952	55373012	-	Eutheria	Eutheria
hsa-miR-502-3p	-1,8	Hsa-Mir-362-P4	hsa-mir-502	MIR-362	MIMAT0002873	MIMAT0004775	chrX	50014612	50014670	+	Eutheria	Eutheria
hsa-miR-10a	-1,7	Hsa-Mir-10-P1a	hsa-mir-10a	MIR-10	MIMAT0000253	MIMAT0004555	chr17	48579864	48579926	-	Vertebrata	Eumetazoa
hsa-miR-27b	-1,7	Hsa-Mir-27-P2	hsa-mir-27b	MIR-27	MIMAT0004588	MIMAT0000419	chr9	95085463	95085525	+	Vertebrata	Vertebrata
hsa-miR-214	-1,7	Hsa-Mir-214	hsa-mir-214	MIR-214	MIMAT0004564	MIMAT0000271	chr1	172138816	172138878	-	Gnathostomata	Gnathostomata
hsa-miR-221	-1,7	Hsa-Mir-221-P1	hsa-mir-221	MIR-221	MIMAT0004568	MIMAT0000278	chrX	45746180	45746242	-	Vertebrata	Vertebrata
hsa-miR-143	-1,7	Hsa-Mir-143	hsa-mir-143	MIR-143	MIMAT0004599	MIMAT0000435	chr5	149428944	149428998	+	Vertebrata	Vertebrata
hsa-miR-370	-1,7	Hsa-Mir-370	hsa-mir-370	MIR-370	MIMAT0026483	MIMAT0000722	chr14	100911151	100911207	+	Boreoeutheria	Boreoeutheria
hsa-miR-139-5p	-1,7	Hsa-Mir-139	hsa-mir-139	MIR-139	MIMAT0000250	MIMAT0004552	chr11	72615066	72615124	-	Gnathostomata	Gnathostomata
hsa-miR-100	-1,7	Hsa-Mir-10-P2a	hsa-mir-100	MIR-10	MIMAT0000098	MIMAT0004512	chr11	122152240	122152296	-	Vertebrata	Eumetazoa
hsa-let-7e	-1,6	Hsa-Let-7-P2	hsa-let-7e	LET-7	MIMAT0000066	MIMAT0004485	chr19	51692793	51692859	+	Vertebrata	Bilateria
hsa-miR-520g	-1,6	Hsa-Mir-430-P37	hsa-mir-520g	MIR-430	MIMAT0026611	MIMAT0002858	chr19	53722182	53722241	+	H. sapiens	Vertebrata
hsa-miR-211	-1,6	Hsa-Mir-204-P2	hsa-mir-211	MIR-204	MIMAT0000268	MIMAT0022694	chr15	31065059	31065116	-	Vertebrata	Vertebrata
hsa-miR-338-5p	-1,6	Hsa-Mir-338-P1	hsa-mir-338	MIR-338	MIMAT0004701	MIMAT0000763	chr17	81125886	81125944	-	Vertebrata	Vertebrata
hsa-miR-520f	-1,5	Hsa-Mir-430-P36	hsa-mir-520f	MIR-430	MIMAT0026609	MIMAT0002830	chr19	53682173	53682233	+	Catarrhini	Vertebrata
hsa-miR-1256	-1,5	Hsa-Mir-1256	hsa-mir-1256	MIR-1256	MIMAT0005907	None	chr1	20988342	20988398	-	Catarrhini	Catarrhini
hsa-miR-328	-1,5	Hsa-Mir-328	hsa-mir-328	MIR-328	MIMAT0026486	MIMAT0000752	chr16	67202327	67202389	-	Eutheria	Eutheria
hsa-miR-1271	-1,5	Hsa-Mir-1271	hsa-mir-1271	MIR-1271	MIMAT0005796	MIMAT0022712	chr5	176367960	176368017	+	Eutheria	Eutheria

Methylation map - 450K

	Gene name ▼	MiRBase ID	Family ▽	5p accession ▽	3p accession ▽	Chromosome	Start	End	Strand	Node of origin (gene) ▽	Node of origin (family) ▽
hsa-miR-1469											
hsa-miR-26B	Hsa-Mir-26-P3	hsa-mir-26b	MIR-26	MIMAT0000083	MIMAT0004500	chr2	218402657	218402713	+	Vertebrata	Vertebrata
hsa-miR-330	Hsa-Mir-330	hsa-mir-330	MIR-330	MIMAT0004693	MIMAT0000751	chr19	45639007	45639070	-	Eutheria	Eutheria
hsa-miR-129–2	Hsa-Mir-129-P2	hsa-mir-129-2	MIR-129	MIMAT0000242	MIMAT0004605	chr11	43581408	43581471	+	Vertebrata	Chordata
hsa-miR-210	Hsa-Mir-210	hsa-mir-210	MIR-210	MIMAT0026475	MIMAT0000267	chr11	568112	568171	-	Bilateria	Bilateria
hsa-miR-1258											
hsa-miR-219–2	Hsa-Mir-219-P2	hsa-mir-219a-2	MIR-219	MIMAT0000276	MIMAT0004675	chr9	128392632	128392696	-	Vertebrata	Bilateria
hsa-miR-10B	Hsa-Mir-10-P1b	hsa-mir-10b	MIR-10	MIMAT0000254	MIMAT0004556	chr2	176150329	176150390	+	Vertebrata	Eumetazoa
hsa-miR-153–2	Hsa-Mir-153-P2	hsa-mir-153-2	MIR-153	MIMAT0026480	MIMAT0000439	chr7	157574347	157574409	-	Vertebrata	Bilateria
hsa-miR-1909											
hsa-miR-1306											
hsa-miR-671	Hsa-Mir-671	hsa-mir-671	MIR-671	MIMAT0003880	MIMAT0004819	chr7	151238449	151238508	+	Theria	Theria
hsa-miR-27b	Hsa-Mir-27-P2	hsa-mir-27b	MIR-27	MIMAT0004588	MIMAT0000419	chr9	95085463	95085525	+	Vertebrata	Vertebrata
hsa-miR-23b	Hsa-Mir-23-P2	hsa-mir-23b	MIR-23	MIMAT0004587	MIMAT0000418	chr9	95085227	95085288	+	Vertebrata	Vertebrata
hsa-miR-24–1	Hsa-Mir-24-P2	hsa-mir-24-1	MIR-24	MIMAT0000079	MIMAT0000080	chr9	95086026	95086085	+	Vertebrata	Vertebrata
hsa-miR-212	Hsa-Mir-132-P2	hsa-mir-212	MIR-132	MIMAT0022695	MIMAT0000269	chr17	2050289	2050350	-	Vertebrata	
hsa-miR-10a	Hsa-Mir-10-P1a	hsa-mir-10a	MIR-10	MIMAT0000253	MIMAT0004555	chr17	48579864	48579926	-	Vertebrata	Eumetazoa
hsa-miR-637											
hsa-miR-339	Hsa-Mir-339	hsa-mir-339	MIR-339	MIMAT0000764	MIMAT0004702	chr7	1022955	1023012	-	Eutheria	Eutheria
hsa-miR-155HG	Hsa-Mir-155	hsa-mir-155	MIR-155	MIMAT0000646	MIMAT0004658	chr21	25573983	25574043	+	Vertebrata	
hsa-miR-1229											
hsa-miR-1276	Hsa-Mir-1276	hsa-mir-1276	MIR-1276	MIMAT0005930	None	chr15	85770508	85770567	-	H. sapiens	H. sapiens
hsa-miR-675	Hsa-Mir-675	hsa-mir-675	MIR-675	MIMAT0004284	MIMAT0006790	chr11	1996766	1996822	-	Eutheria	
hsa-miR-589	Hsa-Mir-589	hsa-mir-589	MIR-589	MIMAT0004799	MIMAT0003256	chr7	5495834	5495894	-	Catarrhini	Catarrhini
hsa-miR-572											
hsa-miR-641	Hsa-Mir-641	hsa-mir-641	MIR-641	MIMAT0003311	None	chr19	40282557	40282626	-	H. sapiens	H. sapiens
hsa-miR-149	Hsa-Mir-149	hsa-mir-149	MIR-149	MIMAT0000450	MIMAT0004609	chr2	240456015	240456076	+	Eutheria	Eutheria
hsa-miR-1224											
hsa-miR-9–3	Hsa-Mir-9-P3	hsa-mir-9-3	MIR-9	MIMAT0000441	MIMAT0000442	chr15	89368032	89368093	+	Vertebrata	Bilateria
hsa-miR-9–1	Hsa-Mir-9-P1	hsa-mir-9-1	MIR-9	MIMAT0000441	MIMAT0000442	chr1	156420353	156420414	-	Vertebrata	Bilateria
hsa-miR-219–1	Hsa-Mir-219-P1	hsa-mir-219a-1	MIR-219	MIMAT0000276	MIMAT0004567	chr6	33207855	33207917	+	Vertebrata	Bilateria
hsa-miR-301B	Hsa-Mir-130-P2b	hsa-mir-301b	MIR-130	MIMAT0032026	MIMAT0004958	chr22	21652990	21653049	+	Vertebrata	Vertebrata
hsa-miR-130B	Hsa-Mir-130-P1b	hsa-mir-130b	MIR-130	MIMAT0004680	MIMAT0000691	chr22	21653316	21653375	+	Vertebrata	Vertebrata
hsa-miR-152	Hsa-Mir-148-P3	hsa-mir-152	MIR-148	MIMAT0026479	MIMAT0000438	chr17	48037174	48037232	-	Vertebrata	Vertebrata
hsa-miR-762											
hsa-miR-193B	Hsa-Mir-193-P1b	hsa-mir-193b	MIR-193	MIMAT0004767	MIMAT0002819	chr16	14303980	14304038	+	Vertebrata	Bilateria
hsa-miR-34b	Hsa-Mir-34-P2a	hsa-mir-34b	MIR-34	MIMAT0000685	MIMAT0004676	chr11	111512951	111513009	+	Vertebrata	Bilateria

hsa-miR-34c-5p	Hsa-Mir-34-P2b	hsa-mir-34c	MIR-34	MIMAT0000686	MIMAT0004677	chr11	111513451	111513505	+	Vertebrata	Bilateria

Table S3 – Putative miR-152 target genes determined by multiple in silico prediction tools.

miRNA	MIMATid	Gene	EntrezID	RefseqID	miRWalk	miRanda	PITA	RNA22	RNAhybrid	Targetscan	SUM
hsa-miR-152-3p	MIMAT0000438	MRPS10	55173	NM_018141	1	1	1	1	1	1	6
hsa-miR-152-3p	MIMAT0000438	C5orf64	285668	NM_173667	1	1	1	1	1	1	6
hsa-miR-152-3p	MIMAT0000438	ATP2A2	488	NM_170665	1	1	1	1	1	1	6
hsa-miR-152-3p	MIMAT0000438	NR2E1	7101	NM_003269	1	1	1	1	1	1	6
hsa-miR-152-3p	MIMAT0000438	SIGLEC8	27181	NM_014442	1	1	1	1	1	1	6
hsa-miR-152-3p	MIMAT0000438	LCOR	84458	NM_032440	1	1	1	1	1	1	6
hsa-miR-152-3p	MIMAT0000438	PPIB	5479	NM_000942	1	1	1	1	1	1	6
hsa-miR-152-3p	MIMAT0000438	PCSK1	5122	NM_000439	1	1	1	1	1	1	6
hsa-miR-152-3p	MIMAT0000438	MAP3K2	10746	NM_006609	1	1	1	1	1	1	6
hsa-miR-152-3p	MIMAT0000438	CUL3	8452	NM_003590	1	1	1	1	1	1	6
hsa-miR-152-3p	MIMAT0000438	NIPA1	123606	NM_144599	1	1	1	1	1	1	6
hsa-miR-152-3p	MIMAT0000438	RASGRP1	10125	NM_005739	1	1	1	1	1	1	6
hsa-miR-152-3p	MIMAT0000438	SLC25A43	203427	NM_145305	1	1	1	1	1	1	6
hsa-miR-152-3p	MIMAT0000438	EPG5	57724	NM_020964	1	1	1	1	1	1	6
hsa-miR-152-3p	MIMAT0000438	CLCN6	1185	NM_001286	1	1	1	1	1	1	6
hsa-miR-152-3p	MIMAT0000438	EZH1	2145	NM_001991	1	1	1	1	1	1	6
hsa-miR-152-3p	MIMAT0000438	RASAL1	8437	NM_004658	1	1	1	1	1	1	6
hsa-miR-152-3p	MIMAT0000438	NUP133	55746	NM_018230	1	1	1	1	1	1	6
hsa-miR-152-3p	MIMAT0000438	UHMK1	127933	NM_175866	1	1	1	1	1	1	6
hsa-miR-152-3p	MIMAT0000438	TMF1	7110	NM_007114	1	1	1	1	1	1	6
hsa-miR-152-3p	MIMAT0000438	PTGER3	5733	NM_198715	1	1	1	1	1	1	6
hsa-miR-152-3p	MIMAT0000438	NPTXR	23467	NM_014293	1	1	1	1	1	1	6
hsa-miR-152-3p	MIMAT0000438	LRRC55	219527	NM_0010052	1	1	1	1	1	1	6
hsa-miR-152-3p	MIMAT0000438	CXCL12	6387	NM_000609	1	1	1	1	1	1	6
hsa-miR-152-3p	MIMAT0000438	KCTD2	23510	NM_015353	1	1	1	1	1	1	6
hsa-miR-152-3p	MIMAT0000438	C1orf95	375057	NM_0010036	1	1	1	1	1	1	6
hsa-miR-152-3p	MIMAT0000438	ADM2	79924	NM_024866	1	1	1	1	1	1	6
hsa-miR-152-3p	MIMAT0000438	LAMB2	3913	NM_002292	1	1	1	1	1	1	6
hsa-miR-152-3p	MIMAT0000438	MEOX2	4223	NM_005924	1	1	1	1	1	1	6

hsa-miR-152-3p	MIMAT0000438	NTN1	9423	NM_004822	1	1	1	1	1	1	6
hsa-miR-152-3p	MIMAT0000438	DNAJC5	80331	NM_025219	1	1	1	1	1	1	6
hsa-miR-152-3p	MIMAT0000438	LGI2	55203	NM_018176	1	1	1	1	1	1	6
hsa-miR-152-3p	MIMAT0000438	YIPF6	286451	NM_173834	1	1	1	1	1	1	6
hsa-miR-152-3p	MIMAT0000438	BMPR2	659	NM_001204	1	1	1	1	1	1	6
hsa-miR-152-3p	MIMAT0000438	TRAPPC10	7109	NM_003274	1	1	1	1	1	1	6
hsa-miR-152-3p	MIMAT0000438	POLM	27434	NM_013284	1	1	1	1	1	1	6
hsa-miR-152-3p	MIMAT0000438	TIGD5	84948	NM_032862	1	1	1	1	1	1	6
hsa-miR-152-3p	MIMAT0000438	PPP2R1B	5519	NM_002716	1	1	1	1	1	1	6
hsa-miR-152-3p	MIMAT0000438	PDE1A	5136	NM_005019	1	1	1	1	1	1	6
hsa-miR-152-3p	MIMAT0000438	ADAMTS5	11096	NM_007038	1	1	1	1	1	1	6
hsa-miR-152-3p	MIMAT0000438	TNFRSF11	8792	NM_003839	1	1	1	1	1	1	6
hsa-miR-152-3p	MIMAT0000438	NR2C2AP	126382	NM_176880	1	1	1	1	1	1	6
hsa-miR-152-3p	MIMAT0000438	SLC7A11	23657	NM_014331	1	1	1	1	1	1	6
hsa-miR-152-3p	MIMAT0000438	SEMA4F	10505	NM_004263	1	1	1	1	1	1	6
hsa-miR-152-3p	MIMAT0000438	ATAD3C	219293	NM_0010392	1	1	1	1	1	1	6
hsa-miR-152-3p	MIMAT0000438	PLEKHA2	59339	NM_021623	1	1	1	1	1	1	6
hsa-miR-152-3p	MIMAT0000438	CLTC	1213	NM_004859	1	1	1	1	1	1	6
hsa-miR-152-3p	MIMAT0000438	FLT1	2321	NM_002019	1	1	1	1	1	1	6
hsa-miR-152-3p	MIMAT0000438	PIK3R3	8503	NM_003629	1	1	1	1	1	1	6
hsa-miR-152-3p	MIMAT0000438	PNPLA2	57104	NM_020376	1	1	1	1	1	1	6
hsa-miR-152-3p	MIMAT0000438	TANGO2	128989	NM_152906	1	1	1	1	1	1	6
hsa-miR-152-3p	MIMAT0000438	TPM3	7170	NM_152263	1	1	1	1	1	1	6
hsa-miR-152-3p	MIMAT0000438	PTGS2	5743	NM_000963	1	1	1	1	1	1	6
hsa-miR-152-3p	MIMAT0000438	FOXK1	221937	NM_0010371	1	1	1	1	1	1	6
hsa-miR-152-3p	MIMAT0000438	SRSF2	6427	NM_003016	1	1	1	1	1	1	6
hsa-miR-152-3p	MIMAT0000438	IL17RA	23765	NM_014339	1	1	1	1	1	1	6
hsa-miR-152-3p	MIMAT0000438	BEND4	389206	NM_207406	1	1	1	1	1	1	6
hsa-miR-152-3p	MIMAT0000438	ERMP1	79956	NM_024896	1	1	1	1	1	1	6
hsa-miR-152-3p	MIMAT0000438	MAF	4094	NM_0010318	1	1	1	1	1	1	6
hsa-miR-152-3p	MIMAT0000438	MMP15	4324	NM_002428	1	1	1	1	1	1	6

hsa-miR-152-3p	MIMAT0000438	SLC4A8	9498	NM_0010399	1	1	1	1	1	1	6
hsa-miR-152-3p	MIMAT0000438	SRCIN1	80725	NM_025248	1	1	1	1	1	1	6
hsa-miR-152-3p	MIMAT0000438	SPTLC3	55304	NM_018327	1	1	1	1	1	1	6
hsa-miR-152-3p	MIMAT0000438	TRIM59	286827	NM_173084	1	1	1	1	1	1	6
hsa-miR-152-3p	MIMAT0000438	TMEM50B	757	NM_006134	1	1	1	1	1	1	6
hsa-miR-152-3p	MIMAT0000438	TNFRSF1B	7133	NM_001066	1	1	1	1	1	1	6
hsa-miR-152-3p	MIMAT0000438	CECR6	27439	NM_031890	1	1	1	1	1	1	6
hsa-miR-152-3p	MIMAT0000438	TANC1	85461	NM_033394	1	1	1	1	1	1	6
hsa-miR-152-3p	MIMAT0000438	PRKX	5613	NM_005044	1	1	1	1	1	1	6
hsa-miR-152-3p	MIMAT0000438	PDE2A	5138	NM_002599	1	1	1	1	1	1	6
hsa-miR-152-3p	MIMAT0000438	KAT7	11143	NM_007067	1	1	1	1	1	1	6
hsa-miR-152-3p	MIMAT0000438	SEMA5A	9037	NM_003966	1	1	1	1	1	1	6
hsa-miR-152-3p	MIMAT0000438	SHE	126669	NM_0010108	1	1	1	1	1	1	6
hsa-miR-152-3p	MIMAT0000438	PTPN11	5781	NM_002834	1	1	1	1	1	1	6
hsa-miR-152-3p	MIMAT0000438	ABHD12	26090	NM_0010424	1	1	1	1	1	1	6
hsa-miR-152-3p	MIMAT0000438	MRPL28	10573	NM_006428	1	1	1	1	1	1	6
hsa-miR-152-3p	MIMAT0000438	ZSCAN25	221785	NM_145115	1	1	1	1	1	1	6
hsa-miR-152-3p	MIMAT0000438	EXOC4	60412	NM_021807	1	1	1	1	1	1	6
hsa-miR-152-3p	MIMAT0000438	CYP2B6	1555	NM_000767	1	1	1	1	1	1	6
hsa-miR-152-3p	MIMAT0000438	GRIK3	2899	NM_000831	1	1	1	1	1	1	6
hsa-miR-152-3p	MIMAT0000438	CDC14A	8556	NM_003672	1	1	1	1	1	1	6
hsa-miR-152-3p	MIMAT0000438	SHROOM3	57619	NM_020859	1	1	1	1	1	1	6
hsa-miR-152-3p	MIMAT0000438	FAM168B	130074	NM_0010099	1	1	1	1	1	1	6
hsa-miR-152-3p	MIMAT0000438	YWHAG	7532	NM_012479	1	1	1	1	1	1	6
hsa-miR-152-3p	MIMAT0000438	SIX4	51804	NM_017420	1	1	1	1	1	1	6
hsa-miR-152-3p	MIMAT0000438	LYSMD2	256586	NM_153374	1	1	1	1	1	1	6
hsa-miR-152-3p	MIMAT0000438	SGCD	6444	NM_000337	1	1	1	1	1	1	6
hsa-miR-152-3p	MIMAT0000438	PARM1	25849	NM_015393	1	1	1	1	1	1	6
hsa-miR-152-3p	MIMAT0000438	IL17REL	400935	NM_0010016	1	1	1	1	1	1	6
hsa-miR-152-3p	MIMAT0000438	LRRTM4	80059	NM_024993	1	1	1	1	1	1	6
hsa-miR-152-3p	MIMAT0000438	MMP10	4319	NM_002425	1	1	1	1	1	1	6

hsa-miR-152-3p	MIMAT0000438	MYO1D	4642	NM_015194	1	1	1	1	1	1	6
hsa-miR-152-3p	MIMAT0000438	SLC25A44	9673	NM_014655	1	1	1	1	1	1	6
hsa-miR-152-3p	MIMAT0000438	SETD7	80854	NM_030648	1	1	1	1	1	1	6
hsa-miR-152-3p	MIMAT0000438	FAM178A	55719	NM_018121	1	1	1	1	1	1	6
hsa-miR-152-3p	MIMAT0000438	NAT8L	339983	NM_178557	1	1	1	1	1	1	6
hsa-miR-152-3p	MIMAT0000438	CHRNB2	1141	NM_000748	1	1	1	1	1	1	6
hsa-miR-152-3p	MIMAT0000438	TRPS1	7227	NM_014112	1	1	1	1	1	1	6
hsa-miR-152-3p	MIMAT0000438	ABI3	51225	NM_016428	1	1	1	1	1	1	6
hsa-miR-152-3p	MIMAT0000438	ANKRD13A	88455	NM_033121	1	1	1	1	1	1	6
hsa-miR-152-3p	MIMAT0000438	RNASEL	6041	NM_021133	1	1	1	1	1	1	6
hsa-miR-152-3p	MIMAT0000438	PDK4	5166	NM_002612	1	1	1	1	1	1	6
hsa-miR-152-3p	MIMAT0000438	BAZ2A	11176	NM_013449	1	1	1	1	1	1	6
hsa-miR-152-3p	MIMAT0000438	LRAT	9227	NM_004744	1	1	1	1	1	1	6
hsa-miR-152-3p	MIMAT0000438	TYW5	129450	NM_0010396	1	1	1	1	1	1	6
hsa-miR-152-3p	MIMAT0000438	PVRL1	5818	NM_002855	1	1	1	1	1	1	6
hsa-miR-152-3p	MIMAT0000438	TMEM97	27346	NM_014573	1	1	1	1	1	1	6
hsa-miR-152-3p	MIMAT0000438	KRR1	11103	NM_007043	1	1	1	1	1	1	6
hsa-miR-152-3p	MIMAT0000438	PCSK9	255738	NM_174936	1	1	1	1	1	1	6
hsa-miR-152-3p	MIMAT0000438	BACH2	60468	NM_021813	1	1	1	1	1	1	6
hsa-miR-152-3p	MIMAT0000438	DPP4	1803	NM_001935	1	1	1	1	1	1	6
hsa-miR-152-3p	MIMAT0000438	HLA-C	3107	NM_002117	1	1	1	1	1	1	6
hsa-miR-152-3p	MIMAT0000438	NOL4	8715	NM_003787	1	1	1	1	1	1	6
hsa-miR-152-3p	MIMAT0000438	ENOPH1	58478	NM_021204	1	1	1	1	1	1	6
hsa-miR-152-3p	MIMAT0000438	AMER1	139285	NM_152424	1	1	1	1	1	1	6
hsa-miR-152-3p	MIMAT0000438	ZNF223	7766	NM_013361	1	1	1	1	1	1	6
hsa-miR-152-3p	MIMAT0000438	PHAX	51808	NM_032177	1	1	1	1	1	1	6
hsa-miR-152-3p	MIMAT0000438	CXorf23	256643	NM_198279	1	1	1	1	1	1	6
hsa-miR-152-3p	MIMAT0000438	SLC5A3	6526	NM_006933	1	1	1	1	1	1	6
hsa-miR-152-3p	MIMAT0000438	TPGS2	25941	NM_015476	1	1	1	1	1	1	6
hsa-miR-152-3p	MIMAT0000438	SNX30	401548	NM_0010129	1	1	1	1	1	1	6
hsa-miR-152-3p	MIMAT0000438	C16orf70	80262	NM_025187	1	1	1	1	1	1	6

hsa-miR-152-3p	MIMAT0000438	MYO5A	4644	NM_000259	1	1	1	1	1	1	6
hsa-miR-152-3p	MIMAT0000438	NDP	4693	NM_000266	1	1	1	1	1	1	6
hsa-miR-152-3p	MIMAT0000438	TBC1D5	9779	NM_014744	1	1	1	1	1	1	6
hsa-miR-152-3p	MIMAT0000438	SYNC	81493	NM_030786	1	1	1	1	1	1	6
hsa-miR-152-3p	MIMAT0000438	BCCIP	56647	NM_078469	1	1	1	1	1	1	6
hsa-miR-152-3p	MIMAT0000438	LIN28B	389421	NM_0010043	1	1	1	1	1	1	6
hsa-miR-152-3p	MIMAT0000438	COL4A1	1282	NM_001845	1	1	1	1	1	1	6
hsa-miR-152-3p	MIMAT0000438	UCP3	7352	NM_003356	1	1	1	1	1	1	6
hsa-miR-152-3p	MIMAT0000438	KRT76	51350	NM_015848	1	1	1	1	1	1	6
hsa-miR-152-3p	MIMAT0000438	PYGO2	90780	NM_138300	1	1	1	1	1	1	6
hsa-miR-152-3p	MIMAT0000438	RPL27A	6157	NM_000990	1	1	1	1	1	1	6
hsa-miR-152-3p	MIMAT0000438	PFAS	5198	NM_012393	1	1	1	1	1	1	6
hsa-miR-152-3p	MIMAT0000438	AKAP11	11215	NM_016248	1	1	1	1	1	1	6
hsa-miR-152-3p	MIMAT0000438	GTPBP1	9567	NM_004286	1	1	1	1	1	1	6
hsa-miR-152-3p	MIMAT0000438	JMY	133746	NM_152405	1	1	1	1	1	1	6
hsa-miR-152-3p	MIMAT0000438	RAG1	5896	NM_000448	1	1	1	1	1	1	6
hsa-miR-152-3p	MIMAT0000438	DLL1	28514	NM_005618	1	1	1	1	1	1	6
hsa-miR-152-3p	MIMAT0000438	ABCB8	11194	NM_007188	1	1	1	1	1	1	6
hsa-miR-152-3p	MIMAT0000438	NEGR1	257194	NM_173808	1	1	1	1	1	1	6
hsa-miR-152-3p	MIMAT0000438	PKNOX2	63876	NM_022062	1	1	1	1	1	1	6
hsa-miR-152-3p	MIMAT0000438	EMP1	2012	NM_001423	1	1	1	1	1	1	6
hsa-miR-152-3p	MIMAT0000438	ITGA5	3678	NM_002205	1	1	1	1	1	1	6
hsa-miR-152-3p	MIMAT0000438	ADAM23	8745	NM_003812	1	1	1	1	1	1	6
hsa-miR-152-3p	MIMAT0000438	ALX4	60529	NM_021926	1	1	1	1	1	1	6
hsa-miR-152-3p	MIMAT0000438	SLFN13	146857	NM_144682	1	1	1	1	1	1	6
hsa-miR-152-3p	MIMAT0000438	PTP4A1	7803	NM_003463	1	1	1	1	1	1	6
hsa-miR-152-3p	MIMAT0000438	ERRFI1	54206	NM_018948	1	1	1	1	1	1	6
hsa-miR-152-3p	MIMAT0000438	SGMS1	259230	NM_147156	1	1	1	1	1	1	6
hsa-miR-152-3p	MIMAT0000438	SNCA	6622	NM_000345	1	1	1	1	1	1	6
hsa-miR-152-3p	MIMAT0000438	CHD5	26038	NM_015557	1	1	1	1	1	1	6
hsa-miR-152-3p	MIMAT0000438	LURAP1	541468	NM_0010136	1	1	1	1	1	1	6

hsa-miR-152-3p	MIMAT0000438	CPEB4	80315	NM_030627	1	1	1	1	1	1	6
hsa-miR-152-3p	MIMAT0000438	MYO5B	4645	NM_0010804	1	1	1	1	1	1	6
hsa-miR-152-3p	MIMAT0000438	NID1	4811	NM_002508	1	1	1	1	1	1	6
hsa-miR-152-3p	MIMAT0000438	ARHGEF17	9828	NM_014786	1	1	1	1	1	1	6
hsa-miR-152-3p	MIMAT0000438	NUDT12	83594	NM_031438	1	1	1	1	1	1	6
hsa-miR-152-3p	MIMAT0000438	TMEM9B	56674	NM_020644	1	1	1	1	1	1	6
hsa-miR-152-3p	MIMAT0000438	NRARP	441478	NM_0010043	1	1	1	1	1	1	6
hsa-miR-152-3p	MIMAT0000438	CPD	1362	NM_001304	1	1	1	1	1	1	6
hsa-miR-152-3p	MIMAT0000438	FZD5	7855	NM_003468	1	1	1	1	1	1	6
hsa-miR-152-3p	MIMAT0000438	AMOTL2	51421	NM_016201	1	1	1	1	1	1	6
hsa-miR-152-3p	MIMAT0000438	SESTD1	91404	NM_178123	1	1	1	1	1	1	6
hsa-miR-152-3p	MIMAT0000438	SELL	6402	NM_000655	1	1	1	1	1	1	6
hsa-miR-152-3p	MIMAT0000438	PHEX	5251	NM_000444	1	1	1	1	1	1	6
hsa-miR-152-3p	MIMAT0000438	SHANK2	22941	NM_012309	1	1	1	1	1	1	6
hsa-miR-152-3p	MIMAT0000438	PPM1F	9647	NM_014634	1	1	1	1	1	1	6
hsa-miR-152-3p	MIMAT0000438	TLDC2	140711	NM_080628	1	1	1	1	1	1	6
hsa-miR-152-3p	MIMAT0000438	RHD	6007	NM_016124	1	1	1	1	1	1	6
hsa-miR-152-3p	MIMAT0000438	TMED7	51014	NM_181836	1	1	1	1	1	1	6
hsa-miR-152-3p	MIMAT0000438	MRPS27	23107	NM_015084	1	1	1	1	1	1	6
hsa-miR-152-3p	MIMAT0000438	SUMF1	285362	NM_182760	1	1	1	1	1	1	6
hsa-miR-152-3p	MIMAT0000438	XPNPEP3	63929	NM_022098	1	1	1	1	1	1	6
hsa-miR-152-3p	MIMAT0000438	EMR1	2015	NM_001974	1	1	1	1	1	1	6
hsa-miR-152-3p	MIMAT0000438	ITPK1	3705	NM_014216	1	1	1	1	1	1	6
hsa-miR-152-3p	MIMAT0000438	NRP1	8829	NM_003873	1	1	1	1	1	1	6
hsa-miR-152-3p	MIMAT0000438	XYLT1	64131	NM_022166	1	1	1	1	1	1	6
hsa-miR-152-3p	MIMAT0000438	RC3H1	149041	NM_172071	1	1	1	1	1	1	6
hsa-miR-152-3p	MIMAT0000438	SF3A2	8175	NM_007165	1	1	1	1	1	1	6
hsa-miR-152-3p	MIMAT0000438	FBXL19	54620	NM_0010997	1	1	1	1	1	1	6
hsa-miR-152-3p	MIMAT0000438	PGM2L1	283209	NM_173582	1	1	1	1	1	1	6
hsa-miR-152-3p	MIMAT0000438	SOX11	6664	NM_003108	1	1	1	1	1	1	6
hsa-miR-152-3p	MIMAT0000438	DNM3	26052	NM_015569	1	1	1	1	1	1	6

hsa-miR-152-3p	MIMAT0000438	WIPF3	644150	NM_0010805	1	1	1	1	1	1	6
hsa-miR-152-3p	MIMAT0000438	CEP44	80817	NM_0010401	1	1	1	1	1	1	6
hsa-miR-152-3p	MIMAT0000438	NOTCH1	4851	NM_017617	1	1	1	1	1	1	6
hsa-miR-152-3p	MIMAT0000438	NPTX1	4884	NM_002522	1	1	1	1	1	1	6
hsa-miR-152-3p	MIMAT0000438	STX6	10228	NM_005819	1	1	1	1	1	1	6
hsa-miR-152-3p	MIMAT0000438	ZNF587	84914	NM_032828	1	1	1	1	1	1	6
hsa-miR-152-3p	MIMAT0000438	NCLN	56926	NM_020170	1	1	1	1	1	1	6
hsa-miR-152-3p	MIMAT0000438	ZNF605	100289635	NM_183238	1	1	1	1	1	1	6
hsa-miR-152-3p	MIMAT0000438	GADD45A	1647	NM_001924	1	1	1	1	1	1	6
hsa-miR-152-3p	MIMAT0000438	USP7	7874	NM_003470	1	1	1	1	1	1	6
hsa-miR-152-3p	MIMAT0000438	INO80	54617	NM_017553	1	1	1	1	1	1	6
hsa-miR-152-3p	MIMAT0000438	OSBPL11	114885	NM_022776	1	1	1	1	1	1	6
hsa-miR-152-3p	MIMAT0000438	SLC1A2	6506	NM_004171	1	1	1	1	1	1	6
hsa-miR-152-3p	MIMAT0000438	PML	5371	NM_033238	1	1	1	1	1	1	6
hsa-miR-152-3p	MIMAT0000438	CMTR1	23070	NM_015050	1	1	1	1	1	1	6
hsa-miR-152-3p	MIMAT0000438	EDEM1	9695	NM_014674	1	1	1	1	1	1	6
hsa-miR-152-3p	MIMAT0000438	STK35	140901	NM_080836	1	1	1	1	1	1	6
hsa-miR-152-3p	MIMAT0000438	ROBO2	6092	NM_002942	1	1	1	1	1	1	6
hsa-miR-152-3p	MIMAT0000438	ST8SIA3	51046	NM_015879	1	1	1	1	1	1	6
hsa-miR-152-3p	MIMAT0000438	TTLL12	23170	NM_015140	1	1	1	1	1	1	6
hsa-miR-152-3p	MIMAT0000438	FAM153A	285596	NM_173663	1	1	1	1	1	1	6
hsa-miR-152-3p	MIMAT0000438	XPO4	64328	NM_022459	1	1	1	1	1	1	6
hsa-miR-152-3p	MIMAT0000438	ERBB3	2065	NM_001982	1	1	1	1	1	1	6
hsa-miR-152-3p	MIMAT0000438	JARID2	3720	NM_004973	1	1	1	1	1	1	6
hsa-miR-152-3p	MIMAT0000438	BSN	8927	NM_003458	1	1	1	1	1	1	6
hsa-miR-152-3p	MIMAT0000438	TRAK2	66008	NM_015049	1	1	1	1	1	1	6
hsa-miR-152-3p	MIMAT0000438	TTL	150465	NM_153712	1	1	1	1	1	1	6
hsa-miR-152-3p	MIMAT0000438	TMEM187	8269	NM_003492	1	1	1	1	1	1	6
hsa-miR-152-3p	MIMAT0000438	VSIG10	54621	NM_019086	1	1	1	1	1	1	6
hsa-miR-152-3p	MIMAT0000438	ZDHHC22	283576	NM_174976	1	1	1	1	1	1	6
hsa-miR-152-3p	MIMAT0000438	ACHE	43	NM_015831	1	1	1	1	1	1	6

hsa-miR-152-3p	MIMAT0000438	SYT4	6860	NM_020783	1	1	1	1	1	1	6
hsa-miR-152-3p	MIMAT0000438	NAT9	26151	NM_015654	1	1	1	1	1	1	6
hsa-miR-152-3p	MIMAT0000438	VANGL1	81839	NM_138959	1	1	1	1	1	1	6
hsa-miR-152-3p	MIMAT0000438	NPC1	4864	NM_000271	1	1	1	1	1	1	6
hsa-miR-152-3p	MIMAT0000438	NRAS	4893	NM_002524	1	1	1	1	1	1	6
hsa-miR-152-3p	MIMAT0000438	IKZF1	10320	NM_006060	1	1	1	1	1	1	6
hsa-miR-152-3p	MIMAT0000438	FAM105B	90268	NM_138348	1	1	1	1	1	1	6
hsa-miR-152-3p	MIMAT0000438	XXYLT1	152002	NM_152531	1	1	1	1	1	1	6
hsa-miR-152-3p	MIMAT0000438	C12orf4	57102	NM_020374	1	1	1	1	1	1	6
hsa-miR-152-3p	MIMAT0000438	AKT2	208	NM_001626	1	1	1	1	1	1	6
hsa-miR-152-3p	MIMAT0000438	S1PR1	1901	NM_001400	1	1	1	1	1	1	6
hsa-miR-152-3p	MIMAT0000438	CUL5	8065	NM_003478	1	1	1	1	1	1	6
hsa-miR-152-3p	MIMAT0000438	HES6	55502	NM_018645	1	1	1	1	1	1	6
hsa-miR-152-3p	MIMAT0000438	MARCH3	115123	NM_178450	1	1	1	1	1	1	6
hsa-miR-152-3p	MIMAT0000438	SLC6A12	6539	NM_003044	1	1	1	1	1	1	6
hsa-miR-152-3p	MIMAT0000438	POU3F2	5454	NM_005604	1	1	1	1	1	1	6
hsa-miR-152-3p	MIMAT0000438	CDK19	23097	NM_015076	1	1	1	1	1	1	6
hsa-miR-152-3p	MIMAT0000438	ST18	9705	NM_014682	1	1	1	1	1	1	6
hsa-miR-152-3p	MIMAT0000438	ROCK1	6093	NM_005406	1	1	1	1	1	1	6
hsa-miR-152-3p	MIMAT0000438	PHF20	51230	NM_016436	1	1	1	1	1	1	6
hsa-miR-152-3p	MIMAT0000438	POFUT2	23275	NM_015227	1	1	1	1	1	1	6
hsa-miR-152-3p	MIMAT0000438	C9orf47	286223	NM_0010019	1	1	1	1	1	1	6
hsa-miR-152-3p	MIMAT0000438	SUDS3	64426	NM_022491	1	1	1	1	1	1	6
hsa-miR-152-3p	MIMAT0000438	HTT	3064	NM_002111	1	1	1	1	1	1	6
hsa-miR-152-3p	MIMAT0000438	LDLR	3949	NM_000527	1	1	1	1	1	1	6
hsa-miR-152-3p	MIMAT0000438	USP6	9098	NM_004505	1	1	1	1	1	1	6
hsa-miR-152-3p	MIMAT0000438	TNIP2	79155	NM_024309	1	1	1	1	1	1	6
hsa-miR-152-3p	MIMAT0000438	C7orf60	154743	NM_152556	1	1	1	1	1	1	6
hsa-miR-152-3p	MIMAT0000438	EPDR1	54749	NM_017549	1	1	1	1	1	1	6
hsa-miR-152-3p	MIMAT0000438	DUSP28	285193	NM_0010335	1	1	1	1	1	1	6
hsa-miR-152-3p	MIMAT0000438	ARSB	411	NM_000046	1	1	1	1	1	1	6

hsa-miR-152-3p	MIMAT0000438	TEAD1	7003	NM_021961	1	1	1	1	1	1	6
hsa-miR-152-3p	MIMAT0000438	B3GAT3	26229	NM_012200	1	1	1	1	1	1	6
hsa-miR-152-3p	MIMAT0000438	SESN2	83667	NM_031459	1	1	1	1	1	1	6
hsa-miR-152-3p	MIMAT0000438	NPTX2	4885	NM_002523	1	1	1	1	1	1	6
hsa-miR-152-3p	MIMAT0000438	OPHN1	4983	NM_002547	1	1	1	1	1	1	6
hsa-miR-152-3p	MIMAT0000438	NEBL	10529	NM_006393	1	1	1	1	1	1	6
hsa-miR-152-3p	MIMAT0000438	PHF21B	112885	NM_138415	1	1	1	1	1	1	6
hsa-miR-152-3p	MIMAT0000438	TECPR2	9895	NM_014844	1	1	1	1	1	1	6
hsa-miR-152-3p	MIMAT0000438	AMZ1	155185	NM_133463	1	1	1	1	1	1	6
hsa-miR-152-3p	MIMAT0000438	PLXDC1	57125	NM_020405	1	1	1	1	1	1	6
hsa-miR-152-3p	MIMAT0000438	ANGPT2	285	NM_001147	1	1	1	1	1	1	6
hsa-miR-152-3p	MIMAT0000438	MEGF9	1955	NM_0010804	1	1	1	1	1	1	6
hsa-miR-152-3p	MIMAT0000438	SLC7A5	8140	NM_003486	1	1	1	1	1	1	6
hsa-miR-152-3p	MIMAT0000438	FOXRED1	55572	NM_017547	1	1	1	1	1	1	6
hsa-miR-152-3p	MIMAT0000438	ZNF526	116115	NM_133444	1	1	1	1	1	1	6
hsa-miR-152-3p	MIMAT0000438	SNRPD1	6632	NM_006938	1	1	1	1	1	1	6
hsa-miR-152-3p	MIMAT0000438	POU4F2	5458	NM_004575	1	1	1	1	1	1	6
hsa-miR-152-3p	MIMAT0000438	LARP4B	23185	NM_015155	1	1	1	1	1	1	6
hsa-miR-152-3p	MIMAT0000438	ATXN1	6310	NM_000332	1	1	1	1	1	1	6
hsa-miR-152-3p	MIMAT0000438	RAB14	51552	NM_016322	1	1	1	1	1	1	6
hsa-miR-152-3p	MIMAT0000438	KIAA1045	23349	NM_015297	1	1	1	1	1	1	6
hsa-miR-152-3p	MIMAT0000438	S100A7A	338324	NM_176823	1	1	1	1	1	1	6
hsa-miR-152-3p	MIMAT0000438	YTHDC2	64848	NM_022828	1	1	1	1	1	1	6
hsa-miR-152-3p	MIMAT0000438	HLA-A	3105	NM_002116	1	1	1	1	1	1	6
hsa-miR-152-3p	MIMAT0000438	LRP2	4036	NM_004525	1	1	1	1	1	1	6
hsa-miR-152-3p	MIMAT0000438	VAPB	9217	NM_004738	1	1	1	1	1	1	6
hsa-miR-152-3p	MIMAT0000438	CCDC170	80129	NM_025059	1	1	1	1	1	1	6
hsa-miR-152-3p	MIMAT0000438	PRICKLE2	166336	NM_198859	1	1	1	1	1	1	6
hsa-miR-152-3p	MIMAT0000438	ELOVL2	54898	NM_017770	1	1	1	1	1	1	6
hsa-miR-152-3p	MIMAT0000438	C3orf70	285382	NM_0010252	1	1	1	1	1	1	6
hsa-miR-152-3p	MIMAT0000438	ZFHX3	463	NM_006885	1	1	1	1	1	1	6

hsa-miR-152-3p	MIMAT0000438	TERF2	7014	NM_005652	1	1	1	1	1	1	6
hsa-miR-152-3p	MIMAT0000438	BBC3	27113	NM_014417	1	1	1	1	1	1	6
hsa-miR-152-3p	MIMAT0000438	RAB34	83871	NM_031934	1	1	1	1	1	1	6
hsa-miR-152-3p	MIMAT0000438	OAS2	4939	NM_002535	1	1	1	1	1	1	6
hsa-miR-152-3p	MIMAT0000438	SERPINE1	5054	NM_000602	1	1	1	1	1	1	6
hsa-miR-152-3p	MIMAT0000438	TXNIP	10628	NM_006472	1	1	1	1	1	1	6
hsa-miR-152-3p	MIMAT0000438	TMEM123	114908	NM_052932	1	1	1	1	1	1	6
hsa-miR-152-3p	MIMAT0000438	DGCR2	9993	NM_005137	1	1	1	1	1	1	6
hsa-miR-152-3p	MIMAT0000438	LONRF2	164832	NM_198461	1	1	1	1	1	1	6
hsa-miR-152-3p	MIMAT0000438	SLC24A3	57419	NM_020689	1	1	1	1	1	1	6
hsa-miR-152-3p	MIMAT0000438	ASTN1	460	NM_004319	1	1	1	1	1	1	6
hsa-miR-152-3p	MIMAT0000438	EPAS1	2034	NM_001430	1	1	1	1	1	1	6
hsa-miR-152-3p	MIMAT0000438	SNN	8303	NM_003498	1	1	1	1	1	1	6
hsa-miR-152-3p	MIMAT0000438	IPO9	55705	NM_018085	1	1	1	1	1	1	6
hsa-miR-152-3p	MIMAT0000438	MED12L	116931	NM_053002	1	1	1	1	1	1	6
hsa-miR-152-3p	MIMAT0000438	STX3	6809	NM_004177	1	1	1	1	1	1	6
hsa-miR-152-3p	MIMAT0000438	PSMD9	5715	NM_002813	1	1	1	1	1	1	6
hsa-miR-152-3p	MIMAT0000438	FBXO28	23219	NM_015176	1	1	1	1	1	1	6
hsa-miR-152-3p	MIMAT0000438	SCN2B	6327	NM_004588	1	1	1	1	1	1	6
hsa-miR-152-3p	MIMAT0000438	PEX5L	51555	NM_016559	1	1	1	1	1	1	6
hsa-miR-152-3p	MIMAT0000438	NUDCD3	23386	NM_015332	1	1	1	1	1	1	6
hsa-miR-152-3p	MIMAT0000438	MYLK4	340156	NM_0010124	1	1	1	1	1	1	6
hsa-miR-152-3p	MIMAT0000438	CDCP1	64866	NM_022842	1	1	1	1	1	1	6
hsa-miR-152-3p	MIMAT0000438	KAL1	3730	NM_000216	1	1	1	1	1	1	6
hsa-miR-152-3p	MIMAT0000438	MXD1	4084	NM_002357	1	1	1	1	1	1	6
hsa-miR-152-3p	MIMAT0000438	CYTH3	9265	NM_004227	1	1	1	1	1	1	6
hsa-miR-152-3p	MIMAT0000438	TRIM45	80263	NM_025188	1	1	1	1	1	1	6
hsa-miR-152-3p	MIMAT0000438	DCP2	167227	NM_152624	1	1	1	1	1	1	6
hsa-miR-152-3p	MIMAT0000438	PTCD3	55037	NM_017952	1	1	1	1	1	1	6
hsa-miR-152-3p	MIMAT0000438	FRYL	285527	NM_015030	1	1	1	1	1	1	6
hsa-miR-152-3p	MIMAT0000438	ATP1A2	477	NM_000702	1	1	1	1	1	1	6

hsa-miR-152-3p	MIMAT0000438	TGFBR1	7046	NM_004612	1	1	1	1	1	1	6
hsa-miR-152-3p	MIMAT0000438	PALD1	27143	NM_014431	1	1	1	1	1	1	6
hsa-miR-152-3p	MIMAT0000438	MAF1	84232	NM_032272	1	1	1	1	1	1	6
hsa-miR-152-3p	MIMAT0000438	PKD2	5311	NM_000297	1	1	1	1	1	1	6
hsa-miR-152-3p	MIMAT0000438	PBX3	5090	NM_006195	1	1	1	1	1	1	6
hsa-miR-152-3p	MIMAT0000438	SLC12A7	10723	NM_006598	1	1	1	1	1	1	6
hsa-miR-152-3p	MIMAT0000438	MRGPRX3	117195	NM_054031	1	1	1	1	1	1	6
hsa-miR-152-3p	MIMAT0000438	ABCF2	10061	NM_007189	1	1	1	1	1	1	6
hsa-miR-152-3p	MIMAT0000438	ANKS6	203286	NM_173551	1	1	1	1	1	1	6
hsa-miR-152-3p	MIMAT0000438	KIAA1210	57481	NM_020721	1	1	1	1	1	1	6
hsa-miR-152-3p	MIMAT0000438	CHUK	1147	NM_001278	1	1	1	1	1	1	6
hsa-miR-152-3p	MIMAT0000438	ETS1	2113	NM_005238	1	1	1	1	1	1	6
hsa-miR-152-3p	MIMAT0000438	FZD6	8323	NM_003506	1	1	1	1	1	1	6
hsa-miR-152-3p	MIMAT0000438	CARKD	55739	NM_018210	1	1	1	1	1	1	6
hsa-miR-152-3p	MIMAT0000438	TMEM52B	120939	NM_153022	1	1	1	1	1	1	6
hsa-miR-152-3p	MIMAT0000438	GCFC2	6936	NM_003203	1	1	1	1	1	1	6
hsa-miR-152-3p	MIMAT0000438	PTGER2	5732	NM_000956	1	1	1	1	1	1	6
hsa-miR-152-3p	MIMAT0000438	ZDHHC17	23390	NM_015336	1	1	1	1	1	1	6
hsa-miR-152-3p	MIMAT0000438	SCN9A	6335	NM_002977	1	1	1	1	1	1	6
hsa-miR-152-3p	MIMAT0000438	RAB6B	51560	NM_016577	1	1	1	1	1	1	6
hsa-miR-152-3p	MIMAT0000438	ZFYVE26	23503	NM_015346	1	1	1	1	1	1	6
hsa-miR-152-3p	MIMAT0000438	ZNF445	353274	NM_181489	1	1	1	1	1	1	6
hsa-miR-152-3p	MIMAT0000438	BHLHE41	79365	NM_030762	1	1	1	1	1	1	6
hsa-miR-152-3p	MIMAT0000438	KRT85	3891	NM_002283	1	1	1	1	1	1	6
hsa-miR-152-3p	MIMAT0000438	MAP1B	4131	NM_005909	1	1	1	1	1	1	6
hsa-miR-152-3p	MIMAT0000438	B4GALT5	9334	NM_004776	1	1	1	1	1	1	6
hsa-miR-152-3p	MIMAT0000438	ULBP1	80329	NM_025218	1	1	1	1	1	1	6
hsa-miR-152-3p	MIMAT0000438	ago/04	192670	NM_017629	1	1	1	1	1	1	6

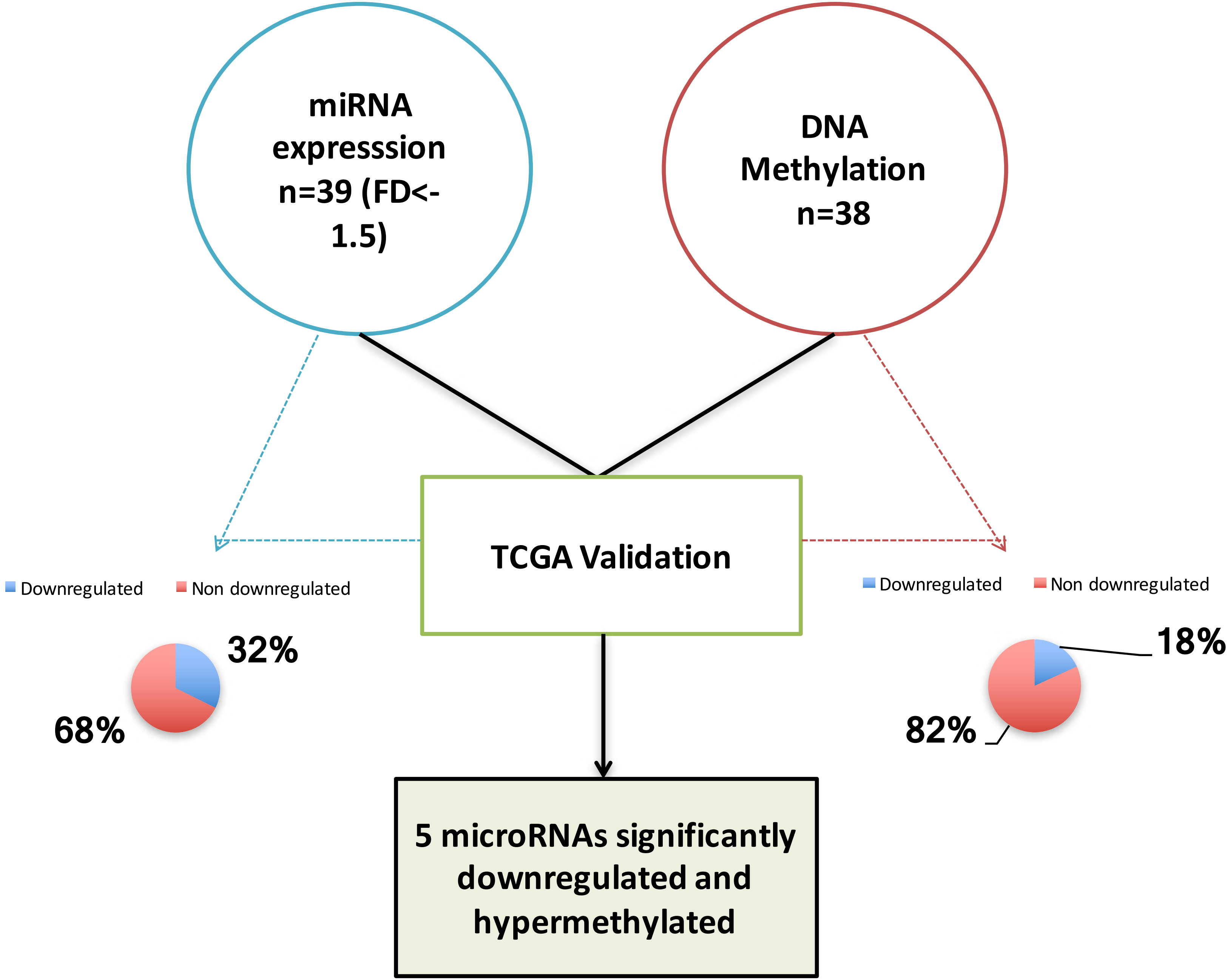
miRNA expresssion n=39 (FD<-1.5)
DNA Methylation n=38
TCGA Validation
Downregulated
Non downregulated
32%
68%
Downregulated
Non downregulated
18%
82%
5 microRNAs significantly downregulated and hypermethylated

Figure S2

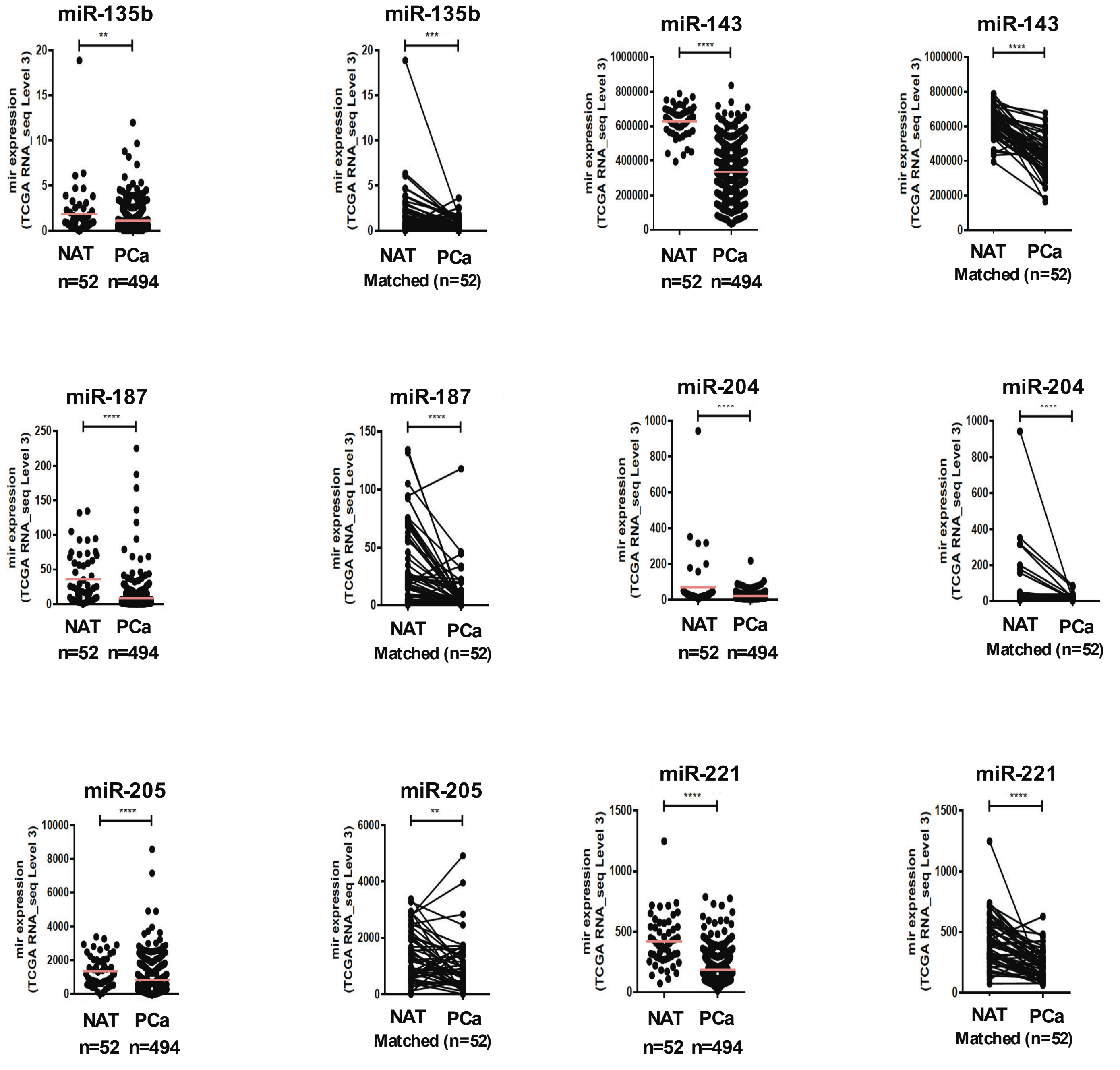
miR-135b
**
mir expression (TCGA RNA_seq Level 3)
NAT n=52
PCa n=494
miR-135b
***
Matched (n=52)
miR-143
****
miR-143
****
miR-187
****
miR-187
****
miR-204
****
miR-204
****
miR-205
****
miR-205
**
miR-221
****
miR-221
****

Figure S3

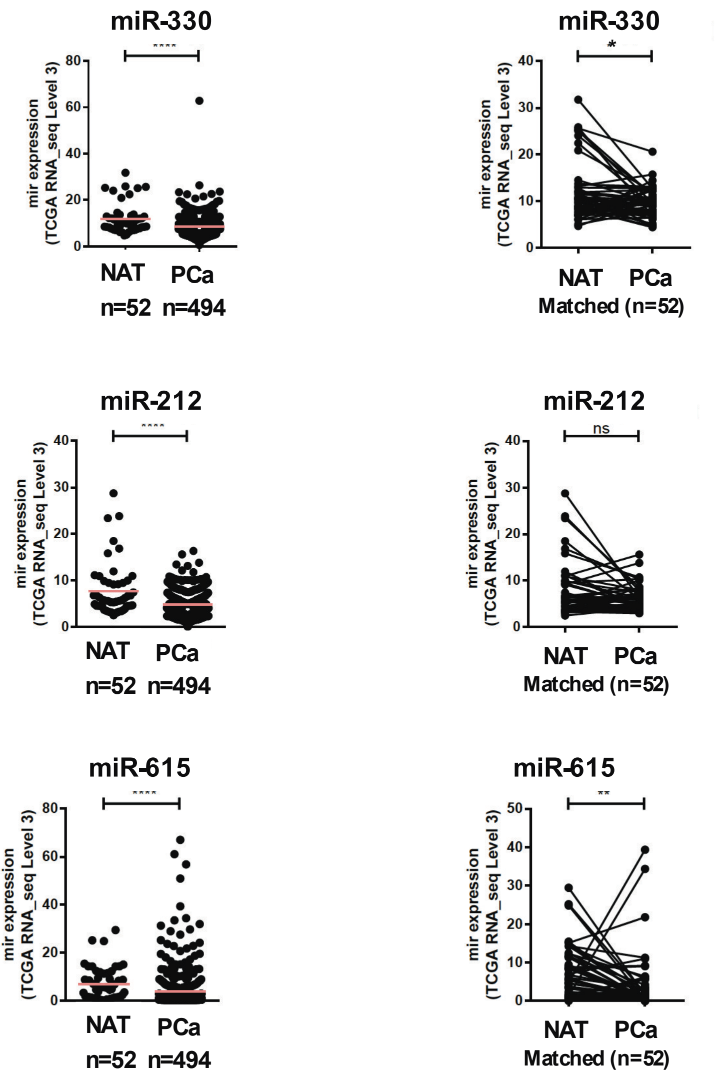
miR-330
****
mir expression
(TCGA RNA_seq Level 3)
80
60
40
20
0
NAT
PCa
n=52
n=494
miR-330
*
mir expression
(TCGA RNA_seq Level 3)
40
30
20
10
0
NAT
PCa
Matched (n=52)
miR-212
****
mir expression
(TCGA RNA_seq Level 3)
40
30
20
10
0
NAT
PCa
n=52
n=494
miR-212
ns
mir expression
(TCGA RNA_seq Level 3)
40
30
20
10
0
NAT
PCa
Matched (n=52)
miR-615
****
mir expression
(TCGA RNA_seq Level 3)
80
60
40
20
0
NAT
PCa
n=52
n=494
miR-615
**
mir expression
(TCGA RNA_seq Level 3)
50
40
30
20
10
0
NAT
PCa
Matched (n=52)

Figure S4

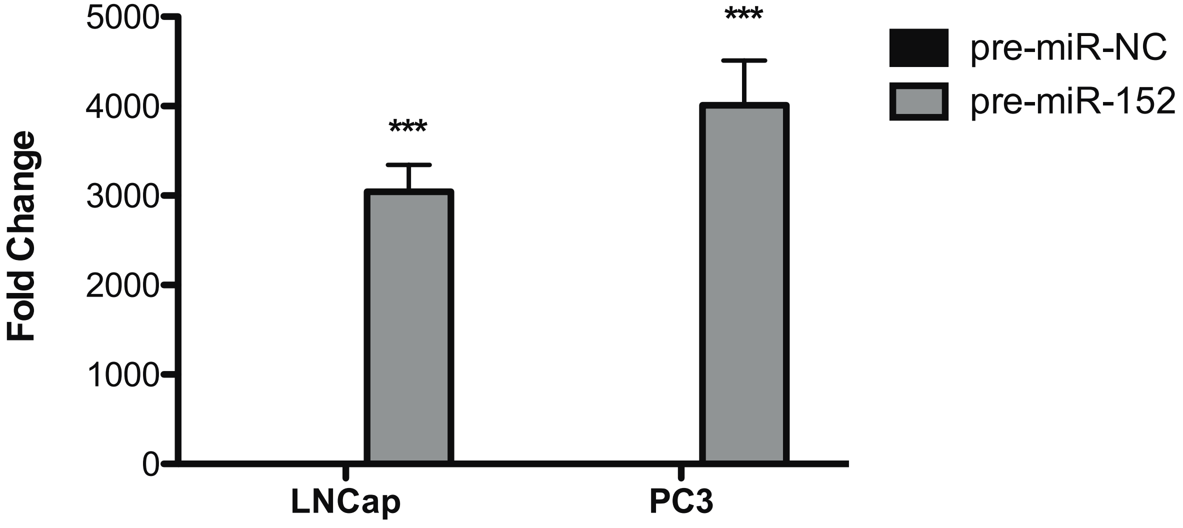

5000
4000
3000
2000
1000
0
Fold Change
***
***
LNCap
PC3
pre-miR-NC
pre-miR-152

Figure S5

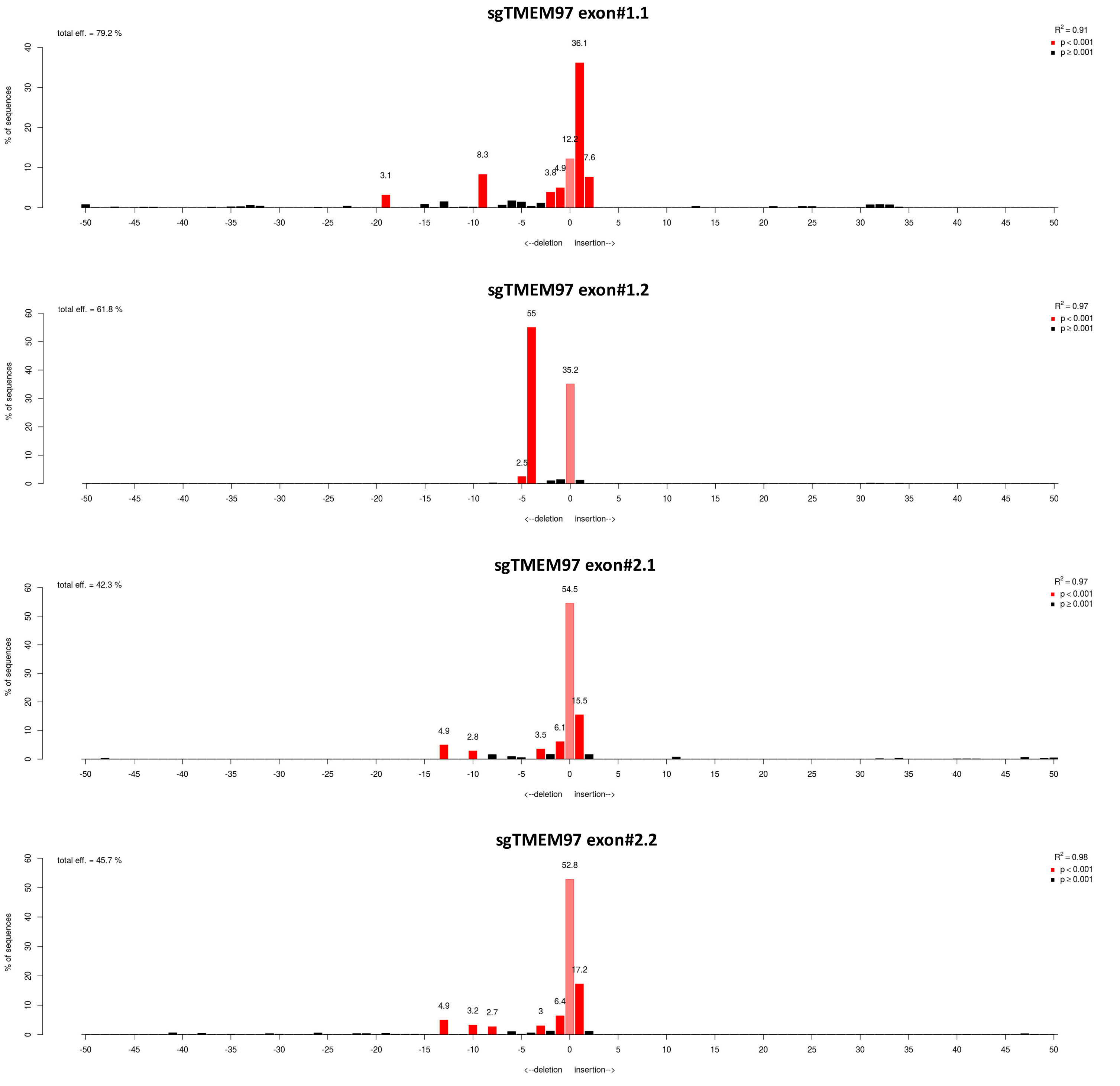
sgTMEM97 exon#1.1
total eff. = 79.2 %
R^2 = 0.91
p < 0.001
p ≥ 0.001
36.1
12.2
8.3
7.6
3.1
3.8
4.9
% of sequences
<--deletion insertion-->
sgTMEM97 exon#1.2
total eff. = 61.8 %
R^2 = 0.97
p < 0.001
p ≥ 0.001
55
35.2
2.5
% of sequences
<--deletion insertion-->
sgTMEM97 exon#2.1
total eff. = 42.3 %
R^2 = 0.97
p < 0.001
p ≥ 0.001
54.5
15.5
4.9
2.8
3.5
6.1
% of sequences
<--deletion insertion-->
sgTMEM97 exon#2.2
total eff. = 45.7 %
R^2 = 0.98
p < 0.001
p ≥ 0.001
52.8
17.2
4.9
3.2
2.7
3
6.4
% of sequences
<--deletion insertion-->
